# Age-dependent regulation of excitatory synaptic transmission at hippocampal temporoammonic-CA1 synapses by leptin

**DOI:** 10.1016/j.neurobiolaging.2018.05.007

**Published:** 2018-09

**Authors:** Gemma McGregor, Leigh Clements, Adham Farah, Andrew J. Irving, Jenni Harvey

**Affiliations:** a Division of Neuroscience, School of Medicine, Ninewells Hospital and Medical School, University of Dundee, Dundee, UK; b School of Biomolecular and Biomedical Science, The Conway Institute, University College Dublin, Dublin, Ireland

**Keywords:** Leptin, Synaptic plasticity, AMPA receptor, Long-term depression, NMDA receptor

## Abstract

The hippocampus is a key target for the hormone leptin and leptin regulation of excitatory synaptic transmission at Schaffer-collateral–CA1 synapses during aging are well documented. However, little is known about the age-dependent actions of leptin at the temporoammonic (TA) input to CA1 neurons. Here we show that leptin induces a novel form of N-methyl-D-aspartate receptor–dependent long-term depression (LTD) at adult (12–24 weeks old) TA-CA1 synapses. Leptin-induced LTD requires activation of canonical Janus tyrosine kinase 2- signal transducer and activator of transcription signaling and removal of GluA1-containing α-amino-3-hydroxy-5-methyl-4-isoxazolepropionic acid receptors from synapses. Moreover, leptin-induced LTD is occluded by activity-dependent LTD at TA-CA1 synapses. By contrast, leptin has no effect on excitatory synaptic transmission at aged (12–14 months old) TA-CA1 synapses, and low-frequency stimulation also fails to induce LTD at this age. These findings demonstrate clear age-related alterations in the leptin sensitivity of TA-CA1 synapses and provide valuable information on how the leptin system alters with age. As leptin has been linked to Alzheimer's disease, these findings have important implications for understanding of age-related disorders such as Alzheimer's disease.

## 1 Introduction

Hippocampal CA1 pyramidal neurons receive 2 distinct glutamatergic inputs. The classical trisynaptic pathway innervates apical dendrites in stratum radiatum via indirect inputs from the CA3 region and the Schaffer-collateral (SC) pathway, whereas the temporoammonic (TA) pathway originates in layer III of the entorhinal cortex (EC) and directly innervates dendrites within the stratum lacunosum-moleculare via the perforant path (Aksoy-Askel and Manahan-Vaughan, 2013). The SC and TA inputs are not only anatomically distinct, but there are also marked differences in the molecular composition of receptors expressed at the 2 inputs (Otmakhova and Lisman, 1999). Moreover, distinct differences in synaptic plasticity mechanisms have been observed at hippocampal SC-CA1 and TA-CA1 synapses (Aksoy-Aksel and Manahan-Vaughan, 2013, McGregor et al., 2017). Growing evidence suggests that TA-CA1 synapses play a fundamental role in spatial novelty detection, intermediate working memory, consolidation, and remote memory retrieval (Remondes and Schuman, 2004, Vago et al., 2007). The TA input is also thought to contribute to the formation of episodic memories by integrating cortical and place cell information (Nakashiba et al., 2008, Remondes and Schuman, 2004). Histological studies indicate that the TA input undergoes significant morphological changes during ageing, and it is an early target in age-related central nervous system disease as degeneration and loss of synapses occur in this pathway in Alzheimer's disease (AD) (Braak and Braak, 1996, Yassa et al., 2010).

It is well established that the anti-obesity hormone leptin regulates food intake and body weight via the hypothalamus (Spiegelman and Flier, 2001). However, leptin receptors are widely expressed in the central nervous system and, numerous studies indicate that leptin targets many brain regions including the hippocampus (Irving and Harvey, 2014). Exposure to leptin results in marked changes in hippocampal synaptic function (Irving and Harvey, 2014, McGregor and Harvey, 2017) and increasing evidence indicates that leptin has cognitive enhancing properties as it facilitates key cellular events underlying hippocampal-dependent learning and memory (Irving and Harvey, 2014, Oomura et al., 2006, Wayner et al., 2004). Recent studies indicate that the modulatory actions of leptin at hippocampal SC-CA1 synapses are highly age dependent. Indeed, during the early stages of postnatal development (P5-8), leptin evokes a persistent depression of excitatory synaptic transmission, long-term depression (LTD), whereas a long-lasting increase long term potentiation (LTP) of synaptic efficacy is induced by leptin in adult (12–16 weeks old) and aged (12–14 months old) hippocampus (Moult and Harvey, 2011). Excitatory synaptic transmission at TA-CA1 synapses is also regulated by this hormone as leptin induces a novel form of N-methyl-D-aspartate (NMDA) receptor–dependent LTP at juvenile (P11-18) TA-CA1 synapses (Luo et al., 2015); an effect that directly opposes the synaptic depression induced by leptin at SC-CA1 synapses at the same stage of development (Moult and Harvey, 2011). However, it is not clear if the ability of leptin to regulate excitatory synaptic transmission at TA-CA1 synapses also alters with age.

Here, we provide the first compelling evidence that in contrast to its effects in juvenile hippocampus, leptin induces a novel form of LTD at adult TA-CA1 synapses. Leptin-induced LTD is likely expressed postsynaptically as it was not associated with any change in the paired pulse facilitation ratio (PPR), and it required selective activation of GluN2A, but not GluN2B, NMDA receptor subunits. Activation of Janus tyrosine kinase 2- signal transducer and activator of transcription (JAK2-STAT3) signaling and subsequent changes in gene transcription underlie leptin-induced LTD, which parallels the proposed role of JAK2-STAT3 signaling in activity-dependent LTD at the same synapse (McGregor et al., 2017). Moreover, leptin-induced LTD is occluded by low-frequency stimulation (LFS)–induced LTD, and vice versa, suggesting a common mechanism of expression underlies both processes. In parallel studies, leptin reduced the surface expression of the α-amino-3-hydroxy-5-methyl-4-isoxazolepropionic acid (AMPA) receptor subunit GluA1 in hippocampal slices and it promoted removal of GluA1 from synapses in hippocampal culture. The leptin-driven internalization of GluA1 is also NMDA receptor–dependent and involves activation of JAK2-STAT3 signaling. In addition, an increase in phosphorylated JAK2 (p-JAK2) and phosphorylated STAT3 (p-STAT3) accompanied leptin-induced LTD and AMPA receptor internalization. By contrast, treatment of aged hippocampal slices with various concentrations of leptin had no significant effect on excitatory synaptic transmission at TA-CA1 synapses. Similarly, application of an LFS paradigm failed to induce LTD at aged TA-CA1 synapses, whereas high-frequency stimulation (HFS) readily resulted in NMDA receptor–dependent LTP at the same synapse. In conclusion, these data indicate that leptin induces a novel form of LTD at adult TA-CA1 synapses. Furthermore, the ability of leptin to regulate synaptic efficacy at TA-CA1 synapses is highly age-dependent as there is marked decline in the leptin sensitivity of TA-CA1 synapses with age. Because dysfunctions in the leptin system are linked to neurodegenerative disorders such as AD, these findings have important implications for the ability of leptin to regulate hippocampal synaptic function in health and age-related disease.

## 2 Materials and methods

### 2.1 Hippocampal slice preparation and electrophysiology

Hippocampal slices (350 μm) were prepared from adult (12–24 weeks old) or aged (12–14 months old) male Sprague-Dawley rats, as previously done (McGregor et al., 2017, Moult and Harvey, 2011). Briefly, animals were killed by deep anesthesia using isoflurane followed by decapitation in accordance with the UK (Scientific Procedures Act) 1986 legislation. Brains were rapidly removed and placed in ice-cold artificial cerebrospinal fluid (aCSF) containing the following (in mM): 124 NaCl, 3 KCl, 26 NaHCO3, 1.25 NaH2PO4, 2 CaCl2, 1 MgSO4, and 10 D-glucose and bubbled with 95% O_2_ and 5% CO_2_. Once prepared, parasagittal hippocampal slices were allowed to recover at room temperature in oxygenated aCSF for 1 hour before use.

As TA-CA1 synapses are electrotonically remote from the CA1 cell somata, standard extracellular recordings of local field excitatory postsynaptic potentials (EPSPs) (fEPSPs) were used to monitor excitatory synaptic transmission at TA-CA1 synapses as previous (Luo et al., 2015, McGregor et al., 2017). In brief, recording pipettes containing aCSF (∼3–5 MΩ) were placed in the stratum lacunosum-moleculare region to record TA-CA1 responses (Otmakhova and Lisman, 1999). The CA3 region was removed from all slices to prevent indirect activation of the trisynaptic pathway. Dopamine (100 μM; 5 minutes) was routinely applied at the end of experiments, to pharmacologically verify stimulation of the TA input (Luo et al., 2015, McGregor et al., 2017). The direct TA pathway was stimulated at 0.033 Hz using a stimulus intensity that evoked peak amplitude ∼50% of the maximum response. Synaptic field potentials were low-pass filtered at 2 kHz and digitally sampled at 10 kHz. Leptin (1–100 nM) was applied to slices for 15 minutes in aCSF, whereas HFS (100 Hz, 1 second) or LFS (1 Hz, 900 shocks, 15 minutes) was used to induce LTP or LTD, respectively.

The slope of the evoked fEPSPs was measured and expressed relative to the preconditioning baseline with 4 consecutive fEPSP slope measurements averaged. Data were monitored online and analyzed offline using the WinLTP program (Anderson and Collingridge, 2007). The degree of leptin or stimulus effect was calculated before the addition of dopamine and expressed as a percentage relative to baseline ±SEM. For antagonist experiments, all slices were preincubated with pharmacological inhibitors 15 minutes before agonist application or stimulus induction. In control experiments, all inhibitors were applied for a minimum of 40 minutes to examine the effect on basal synaptic transmission. Statistical analyses were performed by comparing the 5-minute baseline period before the addition of leptin or stimulus induction protocol or application of pharmacological inhibitor with the 5-minute period before the addition of dopamine using repeated measures analysis of variance. Data was subsequently compared with interleaved control slices using a paired *t*-test. In PPR studies, mean ratios were compared and statistical analysis was conducted using a paired *t*-test (2-tailed; 95% confidence interval). *p* < 0.05 was considered significant with *n* representing the number of slices used from different animals.

### 2.2 Surface biotinylation assay in hippocampal slices

Hippocampal slices (350 μm) from male Sprague-Dawley (12–24 weeks old) rats were obtained as described previously. Slices were incubated with either aCSF (control condition) or aCSF-containing leptin (25 nM; 15 minutes) at room temperature, under continuous gasification with 95% O_2_ and 5% CO_2_. Surface biotinylation and western blotting was carried out as before (McGregor et al., 2017). After treatment, slices were transferred to a slice holder containing 100 μM NHS-SS-biotin (Thermo Fisher, UK) in aCSF, bubbled with 95% O_2_ and 5% CO_2_ for 1 hour. Hippocampi were dissected and washed 3 times with aCSF containing 10 mM glycine and homogenized in 800 μL ice-cold lysis buffer containing the following (in mM): 20 Tris HCl, 50 NaCl, 1 EGTA, 0.1% sodium dodecyl sulfate and 1% Triton X-100 supplemented with a cocktail of protease inhibitors (Roche, Germany). After incubation on ice for 30 minutes, homogenates were centrifuged for 10 minutes at 13,000 rpm, and supernatants were collected. Protein concentration was quantified using a Bradford Assay (VWR, UK). Supernatants from individual slices were combined such that precipitates used for biotinylation were from 5 individual slices, leptin treated (*n* = 5) or control (*n* = 5). About 500 μg of total protein lysate for each condition was precipitated with 60 μL of UltraLink Streptavadin agarose beads (Thermo Fisher, UK) on a rotator overnight at 4 °C. Nonbiotinylated protein was removed by centrifugation (14,000 rpm; 1 minute), and beads were washed 3 times with 500 μL of lysis buffer. Biotinylated protein was eluted with 1% mercaptoethanol denaturing buffer and boiled at 95 °C for 5 minutes. Proteins eluted from beads were subjected to sodium dodecyl sulphate–polyacrylamide gel electrophoresis for 1 hour and transferred to a nitrocellulose membrane for 2 hours. To reduce nonspecific protein binding, 5% rabbit serum (Sigma, UK) was applied for 1 hour. Blots were then treated with an antibody against the N-terminus of GluA1 (sheep anti-GluA1; in-house antibody against synthetic peptide [RTSDSRDHTRVDWKR] corresponding to residues 253–267 of GluA1; 1:750; Moult et al., 2010) overnight at 4 °C. Blots were then washed for 1 hour in tris-buffered saline tween (1.8 M NaCl, 80 mM Tris Base and 1.6 M Tween 20), followed by a 45-minute wash in 5% rabbit serum (Sigma, UK) then incubated with horseradish peroxidase rabbit antisheep secondary antibody (1:2000) for 1 hour at room temperature. Finally, blots were washed for 1.5 hours with tris-buffered saline tween. Chemiluminescent images were obtained using Bio-Rad ChemiDoc XRS imaging system and band density was quantified using the Quantity One software package (Bio-Rad).

### 2.3 Hippocampal cell culture

Hippocampal cultures were prepared as previously (Moult et al., 2010). Briefly, neonatal Sprague-Dawley rats (1–3 days old) were killed by cervical dislocation in accordance with Schedule 1 of UK Animals (Scientific Procedures Act, 1986). Hippocampi were removed, and after washing in HEPES-buffered saline comprising (in mM) 135 NaCl, 5 KCl, 1 CaCl_2_, 1 MgCl_2_, 10 HEPES, and 25 D-glucose (pH 7.4), the hippocampi were treated with papain (1.5 mgml-1; Sigma Aldrich, UK) for 20 minutes at 37 °C. Dissociated cells were plated onto sterile dishes (35 mm diameter; Greiner Bio-One Ltd., UK) treated with poly-d-lysine (20 μgml-1; 1–2 hours). Cultures were maintained in serum replacement medium (SR2; Sigma, UK) in a humidified atmosphere of 95% O_2_ and 5% CO_2_ at 37 °C for up to 2 weeks.

### 2.4 Immunocytochemistry

Immunocytochemistry was performed on 8- to 12-day-old cultured hippocampal neurons. Before labeling, neurons were washed with HEPES buffered saline containing glycine (0.01 mM) and treated with leptin (50 nM) for 30 minutes at 21–23 °C. For antagonist experiments, neurons were pretreated with pharmacological inhibitors for 30 minutes before leptin treatment. All experiments were performed under conditions of low Mg^2+^ (0.1 mM). To label surface GluA1, living neurons were incubated with an antibody against the N-terminal region of GluA1 (sheep anti-GluA1; 1:100; Moult et al., 2010) at 4 °C. Neurons were then fixed with 4% paraformaldehyde for 5 minutes. Surface GluA1 immunostaining was visualized by addition of an appropriate anti-sheep Alexa 488–conjugated secondary antibody (1:250; Life Technologies, UK) for 30 minutes. In a subset of experiments, neurons were permeabilized with 0.1% Triton X-100 (5 minutes) after GluA1 immunolabeling and fixed with 4% paraformaldehyde. A second primary antibody was then applied to compare GluA1 surface immunostaining relative to p-JAK2 (rabbit anti-JAK2 phospho Y1007-1008; 1:250; Abcam, UK), p-STAT3 (rabbit anti-STAT3 phospho Y705; 1:500; Abcam, UK), or PSD-95 (mouse anti-PSD-95; 1:500; Thermo Fisher, UK). p-JAK2 and p-STAT3 staining was visualized by addition of an anti-rabbit Alexa 555–conjugated secondary antibody (1:250; Life Technologies, UK) for 30 minutes, whereas an Alexa 569–conjugated anti-mouse secondary antibody (1:200; Thermo Fisher, UK) was used to visualize PSD-95 labeling (McGregor et al., 2017). No labeling was observed after incubation with secondary antibodies alone.

### 2.5 Analysis

A confocal imaging system (Zeiss LSM 510) was used for image acquisition, and 488-nm and 543-nm laser lines were used to excite the Alexa 488 and 555 or 568 fluorophores, respectively. Images were obtained in a single-tracking mode or multi-tracking mode for dual labeling experiments using a 15-second scan speed. Intensity of staining was determined offline using LaserSharp software (Carl Zeiss). Analysis lines (50 μm) were drawn along randomly selected dendritic regions and mean fluorescence intensity for GluA1, p-JAK2 or p-STAT3 surface staining was calculated for each dendrite (Malekizadeh et al., 2017, McGregor et al., 2017, Moult et al., 2010). For synaptic co-localization experiments, surface GluA1 immunolabeling was compared with dendritic PSD-95 immunostaining. The number of GluA1-positive sites that co-localized with PSD-95–positive sites were counted and expressed as a percentage of the number of PSD-95–positive sites (McGregor et al., 2017). Data were obtained from at least 3 dendrites from a minimum of 4 randomly selected neurons for each treatment, and all data were obtained from at least 3 different cultures from different animals. Within a given experiment, all conditions, including illumination intensity and photomultiplier gains, were kept constant. To quantify experimental data obtained from separate days, data were normalized relative to mean fluorescence intensity in control neurons. For antagonist experiments, neurons treated with leptin in the presence of a pharmacological inhibitor were normalized to data obtained from neurons treated with the inhibitor alone. All data are expressed as means ±SEM, and statistical analyses were performed using 1-way analysis of variance for comparisons between multiple groups. *p* < 0.05 was considered significant with *n* representing the number of labeled dendrites analyzed across all experiments.

## 3 Results

### 3.1 Leptin evokes a persistent decrease in excitatory synaptic transmission at adult TA-CA1 synapses

**Fig. 1:**
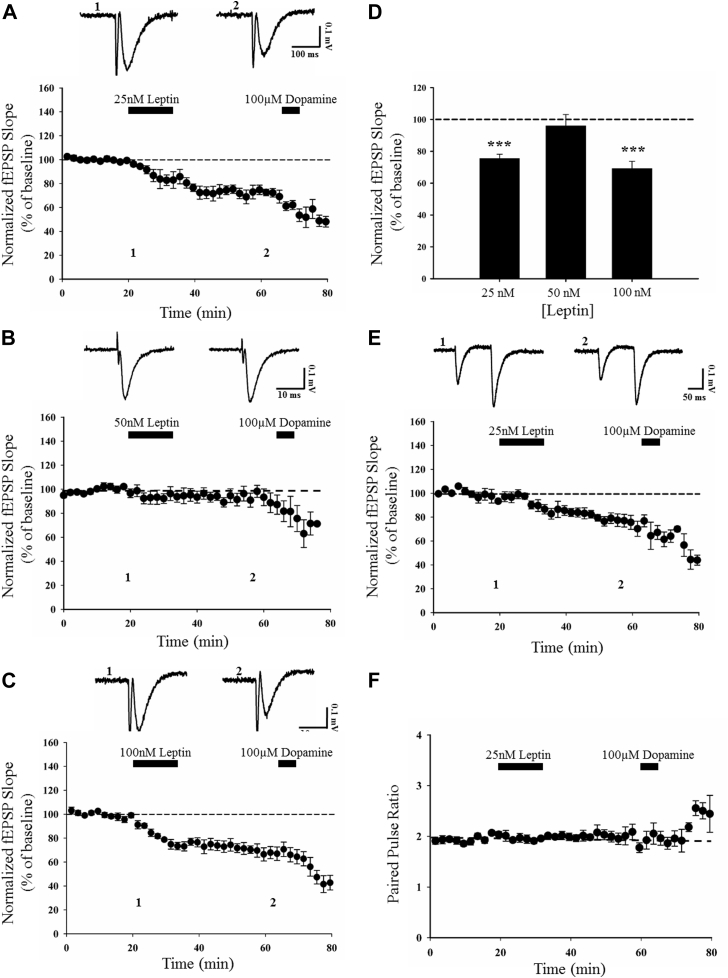
Leptin induces LTD via a postsynaptic expression mechanism at adult TA-CA1 synapses. (A–C) Pooled and normalized data showing the effects of various concentrations of leptin (25–100 nM; 15 minutes) on excitatory synaptic transmission at TA-CA1 synapses in adult (12–24 weeks old) hippocampal slices. Application of dopamine (100 μM; 5 minutes) rapidly inhibits synaptic transmission, confirming stimulation of the TA input. In this and subsequent figures, each point is the average of 4 successive responses, and representative synaptic traces for each experiment are shown above each plot and for the time indicated. Application of 25 nM (A) or 100 nM leptin (C) induced a persistent depression of excitatory synaptic transmission, whereas 50 nM leptin (B) was without effect. (D) Histogram of pooled data illustrating the concentration-dependent effects of leptin. (E, F) Plots of pooled data demonstrating that leptin-induced LTD (E) is not accompanied by alterations in the PPR (F) indicating a postsynaptic expression mechanism. Data in (F) corresponds to mean PPR against time plotted from experiments in (E); top: representative pairs of fEPSPs evoked with a 50-ms interstimulus interval. In this and subsequent figures, *, **, and *** represent *p* < 0.05, *p* < 0.01, and *p* < 0.001, respectively. Abbreviations: LTD, long-term depression; PPR, paired pulse facilitation ratio; TA, temporoammonic.

Previous studies indicate that leptin induces distinct age-dependent effects on excitatory synaptic transmission at SC-CA1 synapses. Indeed, leptin induces LTP in excitatory synaptic transmission at adult hippocampal SC-CA1 synapses (Moult et al., 2010), whereas a synaptic depression is induced by leptin at the same synapse in juvenile hippocampus (Moult and Harvey, 2011). Our recent studies indicate that in contrast to its actions at SC-CA1 synapses, leptin induces a novel form of NMDA receptor–dependent LTP at the anatomically distinct TA input to CA1 neurons (Luo et al., 2015). However, the effects of leptin at adult TA-CA1 synapses are not clear. Thus, to examine this, a range of concentrations of leptin (25–100 nM; 15 minutes) was directly applied to acute hippocampal slices from adult rats (12–24 weeks old) and the effects on excitatory synaptic transmission were assessed. Application of 25 nM leptin resulted in a marked reduction in synaptic transmission to 63 ± 4.4% of baseline (*n* = 7; *p* < 0.01; Fig. 1A, D) that persisted for the duration of recordings up to 30 minutes after leptin washout. By contrast, treatment with an intermediate concentration of leptin (50 nM; 15 minutes) had no significant effect on the magnitude of excitatory synaptic transmission (94 ± 5.5% of baseline; *n* = 7; *p* > 0.05; Fig. 1B, D). However, application of a higher concentration of leptin (100 nM; 15 minutes) resulted in a long-term reduction in synaptic transmission (LTD; 68 ± 5.1% of baseline; *n* = 7; *p* < 0.001; Fig. 1C, D), that persisted after leptin washout. As previous studies indicate that dopamine depresses excitatory synaptic transmission at TA-CA1 synapses, but not SC-CA1 synapses (Otmakhova and Lisman, 1999), dopamine was routinely applied at the end of experiments to pharmacologically verify that recordings were from TA-CA1 synapses (Luo et al., 2015, McGregor et al., 2017). These data indicate that leptin induces a novel form of LTD at adult TA-CA1 synapses, and this follows a clear hormetic concentration-dependent profile.

### 3.2 Leptin-induced LTD has a postsynaptic locus of expression

To characterize leptin-induced LTD further, in all subsequent experiments, LTD was induced by addition of 25 nM leptin for 15 minutes. Previous studies have detected high levels of leptin receptor expression at both presynaptic and postsynaptic sites on hippocampal neurons (Shanley et al., 2002). Consequently, leptin receptors located at either site could contribute to leptin-induced LTD at adult TA-CA1 synapses. Thus, to identify the locus of leptin-induced LTD, the PPR was monitored during experiments as alterations in PPR classically reflect alterations in the probability of glutamate release (Pr). Application of leptin resulted in a persistent depression of synaptic transmission to 75 ± 5.0% of baseline (*n* = 6; *p* < 0.05); however, this was not accompanied by any significant alteration in the PPR (*n* = 6; *p* > 0.05 using a paired *t*-test; Fig. 1E, F). These data suggest that leptin-induced LTD at adult TA-CA1 synapses involves a postsynaptic expression mechanism.

### 3.3 Leptin-induced LTD involves GluN2A-containing NMDA receptors

**Fig. 2:**
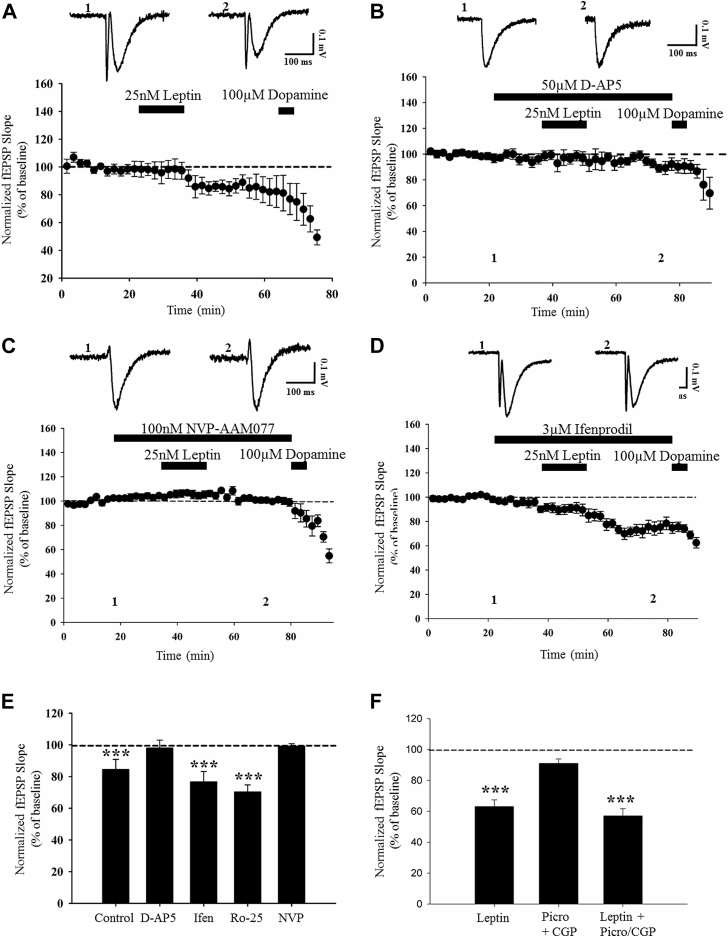
Leptin-induced LTD is GluN2A-NMDA receptor dependent. (A-D) Plots of pooled and normalized data illustrating the effects of different NMDA receptor antagonists on leptin-induced LTD in adult hippocampal slices. In control slices, application of leptin (25 nM, 15 minutes) induced robust LTD (A). In slices treated with the NMDA receptor antagonist, D-AP5 (50 μM; [B]), or the GluN2A-selective antagonist, NVP-AAM077 (100 nM; [C]), the ability of leptin to induce LTD was inhibited. Conversely, the GluN2B antagonist, ifenprodil (3 μM; [D]), failed to inhibit leptin-induced LTD. Each point is the average of four successive responses and representative synaptic traces for each experiment are shown above each plot and for the time indicated. (E) Histogram of pooled data illustrating the relative effects of leptin on synaptic transmission in control conditions and in the presence of D-AP5, NVP-AMM077, ifenprodil, or the GluN2B antagonist, Ro-256981 (3 μM). In this and subsequent histograms, statistical analyses compared the effect of leptin in the presence of pharmacological inhibitor relative to control. GluN2A-containing, but not GluN2B-containing, NMDA receptors are required for leptin-induced LTD at adult TA-CA1 synapses. (F) Histogram of pooled data illustrating the relative effects of leptin on synaptic transmission in control conditions and in the presence of picrotoxin and CGP55854 to block GABAergic inhibition. Leptin-induced LTD does not involve activation of GABA_A_ and GABA_B_ receptors. In this and subsequent figures, *, **, and *** represent *p* < 0.05, *p* < 0.01, and *p* < 0.001, respectively. Abbreviations: GABA, gamma-aminobutyric acid; LTD, long-term depression.

The synaptic activation of NMDA receptors is critical for the induction of activity-dependent LTD at SC-CA1 synapses (Collingridge et al., 2010). Our previous studies indicate NMDA receptor activation is also pivotal for leptin-driven changes in excitatory synaptic efficacy at SC-CA1 synapses (Durakoglugil et al., 2005, Irving and Harvey, 2014, Shanley et al., 2001). Moreover, the ability of leptin to induce LTP at juvenile TA-CA1 synapses also requires activation of NMDA receptors (Luo et al., 2015). Thus, to explore the possible role of NMDA receptors, the effects of the competitive NMDA receptor antagonist, D-AP5 were examined. Application of D-AP5 (50 μM; 40 minutes) had no effect on basal excitatory synaptic transmission (104 ± 3.9% of baseline; *n* = 4; *p* > 0.05; not shown). However, in slices treated with D-AP5, the ability of leptin to induce LTD was blocked such that leptin failed to depress synaptic transmission (95 ± 5.5% of baseline; *n* = 6; *p* > 0.05; Fig. 2B, E) in the presence of D-AP5—an effect that was significantly different (*p* < 0.05; Fig. 2E) from the robust LTD induced by leptin (84 ± 6.3% of baseline; *n* = 5; *p* < 0.001; Fig. 2A, E) in control slices. These data indicate that NMDA receptor activation is required for leptin-induced LTD at adult TA-CA1 synapses.

Molecularly distinct NMDA receptors are involved in different forms of activity-dependent hippocampal synaptic plasticity (Bartlett et al., 2007, Liu et al., 2004). The leptin-driven alterations in synaptic efficacy at SC-CA1 and TA-CA1 synapses also depend on distinct NMDA receptor GluN2 subunits (Luo et al., 2015, Moult and Harvey, 2011). Thus, to verify if specific GluN2 subunits mediate leptin-induced LTD at TA-CA1 synapses, the role of GluN2B subunits was initially examined using 2 distinct inhibitors of GluN2B, namely ifenprodil (3 μM; Williams, 1993) and Ro-256981 (3 μM; Fischer et al., 1997). In control slices, application of either inhibitor for 40 minutes had no effect on basal synaptic transmission (ifenprodil; 102 ± 6.5% of baseline; *n* = 4; *p* > 0.05 and Ro-256981; 96 ± 9.4% of baseline; *n* = 4; *p* > 0.05; not shown). Moreover, application of leptin to control slices resulted in a persistent reduction in synaptic efficacy to 84 ± 6.3% of baseline (*n* = 5; *p* < 0.001; Fig. 2A, E). However, application of leptin also resulted in robust LTD in interleaved slices treated with either ifenprodil (78 ± 3.8% of baseline; *n* = 7; *p* < 0.001; Fig. 2D, E) or Ro-256981 (70 ± 4.4% of baseline; *n* = 6; *p* < 0.001; Fig. 2E), suggesting that leptin-induced LTD is independent of GluN2B subunits. To explore the possible role of GluN2A subunits, the effects of the putative GluN2A antagonist, NVP-AAM077 were examined (Frizelle et al., 2006). Treatment of slices with NVP-AAM077 (100 nM; 40 minutes) had no effect on basal synaptic transmission (95 ± 3.4% of baseline *n* = 4; *p* > 0.05; not shown). By contrast, in slices exposed to NVP-AAM077, the ability of leptin to induce LTD was completely blocked such that synaptic transmission was 103 ± 1.9% of baseline (*n* = 7; *p* > 0.05; Fig. 2C, E) after application of leptin, and this effect was significantly different to the magnitude of LTD induced by leptin in control conditions (*p* < 0.05; Fig. 2E). Thus, these data indicate that the ability of leptin to induce LTD at adult TA-CA1 synapses requires the activation of GluN2A-containing NMDA receptors.

As previous studies have shown that stimulation of the TA pathway can activate gamma-aminobutyric acid (GABA)ergic interneurons, which synapse onto CA1 pyramidal cells (Dvorak-Carbone and Schuman, 1999), it is feasible that GABAergic inhibition plays a role in leptin-induced LTD. To explore this, the ability of leptin to induce LTD was examined in slices treated with antagonists for GABA_A_, (picrotoxin; 50 μM) and GABA_B_ (CGP55845; 100 nM) receptors. In control slices treated with both antagonists, no significant alteration in basal excitatory synaptic transmission was observed (91 ± 2.9% of baseline; *n* = 5; *p* > 0.05). In addition, application of leptin (25 nM; 15 minutes) in the absence of the GABA receptor antagonists resulted in robust LTD as synaptic transmission was depressed to 63 ± 4.4% of baseline (*n* = 5; *p* < 0.001; Fig. 2F). Moreover, the ability of leptin to induce LTD was unaffected when GABAergic inhibition was blocked. Thus in slices treated with a combination of picrotoxin and CGP55845, subsequent application of leptin (25 nM; 15 minutes) resulted in a persistent depression of synaptic transmission to 57 ± 4.7% of baseline (*n* = 5; *p* < 0.001; Fig. 2F). These data indicate that leptin-induced LTD at TA-CA1 synapses is independent of GABAA or GABAB receptor activation.

### 3.4 A role for JAK in leptin-induced LTD

**Fig. 3:**
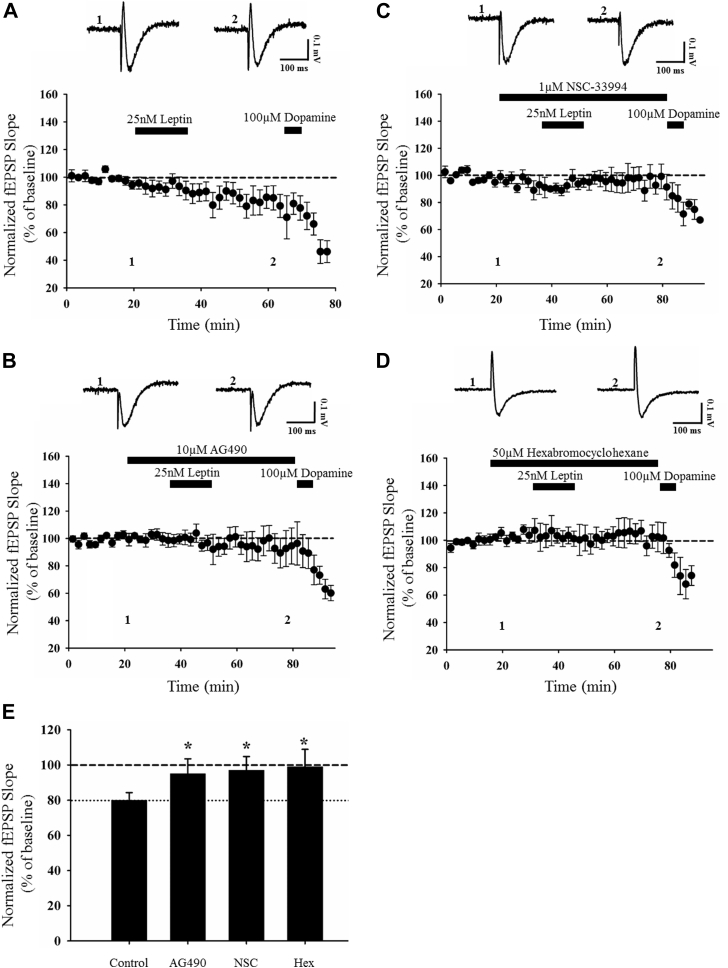
JAK2 activation is required for leptin-induced LTD. (A–D) Plots of pooled and normalized data illustrating the effects of leptin on synaptic transmission in control conditions (A) and in the presence of the broad spectrum JAK inhibitor, AG490 (10 μM; [B]), or specific JAK2 inhibitors, NSC-33994 (1 μM; [C]) and hexabromocyclohexane (50 μM; [D]). (E) Histogram of pooled data demonstrating the relative effects of leptin (25 nM) on excitatory synaptic transmission in control conditions and in the presence of AG490, NSC-33994, or hexabromocyclohexane. Leptin-induced LTD involves the activation of JAK2. In this and subsequent figures, *, **, and *** represent *p* < 0.05, *p* < 0.01, and *p* < 0.001, respectively. Abbreviations: LTD, long-term depression.

Recent studies indicate that JAK-STAT signaling is pivotal for activity-dependent LTD at hippocampal SC-CA1 synapses (Nicolas et al., 2012), and at adult TA-CA1 synapses (McGregor et al., 2017). Thus, as the JAK-STAT pathway is activated by leptin receptors, it is feasible that this pathway also contributes to leptin-induced LTD at TA-CA1 synapses. Thus, to establish if JAKs play a role, the effects of a broad spectrum JAK inhibitor, AG490 were evaluated (Meydan et al., 1996). Treatment of hippocampal slices with AG490 (10 μM; 40 minutes) had no effect on basal synaptic transmission (107 ± 6.5% of baseline; *n* = 4; *p* > 0.05; not shown). Furthermore, application of leptin (25 nM; 15 minutes) resulted in robust LTD as synaptic transmission was reduced to 80 ± 4.3% of baseline in control slices (*n* = 5; *p* < 0.05; Fig. 3A, E). However, in interleaved slices exposed to AG490 (10 μM), leptin failed to induce LTD such that synaptic transmission was 95 ± 8.5% of baseline (*n* = 6; *p* > 0.05) after exposure to leptin (Fig. 3B, E), and this effect was significantly different to the magnitude of leptin-induced LTD in control conditions (*p* < 0.05; Fig. 3E). Thus, these data indicate that JAK activation plays a key role in leptin-induced LTD at adult TA-CA1 synapses.

Although 4 isoforms of JAK have been identified, only the JAK2 isoform is enriched at hippocampal synapses and is implicated in hippocampal LTD at SC-CA1 and TA-CA1 synapses (McGregor et al., 2017, Nicolas et al., 2012). Thus, to explore the possible role of JAK2, 2 different inhibitors of JAK2, namely NSC-33994 and hexabromocyclohexane (Sandberg et al., 2005), were used. Application of either hexabromocyclohexane (50 μM) or NSC-33994 (1 μM) for 40 minutes had no significant effect on basal synaptic transmission (hexabromocyclohexane; 97 ± 8.6% of baseline *n* = 4; *p* > 0.05 and NSC-33994; 99 ± 12.0% of baseline; *n* = 4; *p* > 0.05; not shown). However, in slices treated with either JAK2 inhibitor, the ability of leptin to induce LTD was significantly reduced as synaptic transmission was 97 ± 7.8% of baseline (NSC-33994; *n* = 5; *p* > 0.05) and 99 ± 9.9% of baseline (hexabromocyclohexane; *n* = 6; *p* > 0.05) after leptin application, respectively (Fig. 3C–E). By contrast, robust LTD (80 ± 4.3% of baseline; *n* = 5; *p* < 0.05; Fig. 3A, E) was induced by leptin (25 nM; 15 minutes) in interleaved control slices, and this effect was significantly different to leptin's effect in slices exposed to NSC-33994 or hexabromocyclohexane (*p* < 0.05 for each; Fig. 3E). These data indicate that the ability of leptin to induce LTD at adult TA-CA1 synapses requires the activation of JAK2.

### 3.5 STAT3 signaling plays a crucial role in leptin-induced LTD

**Fig. 4:**
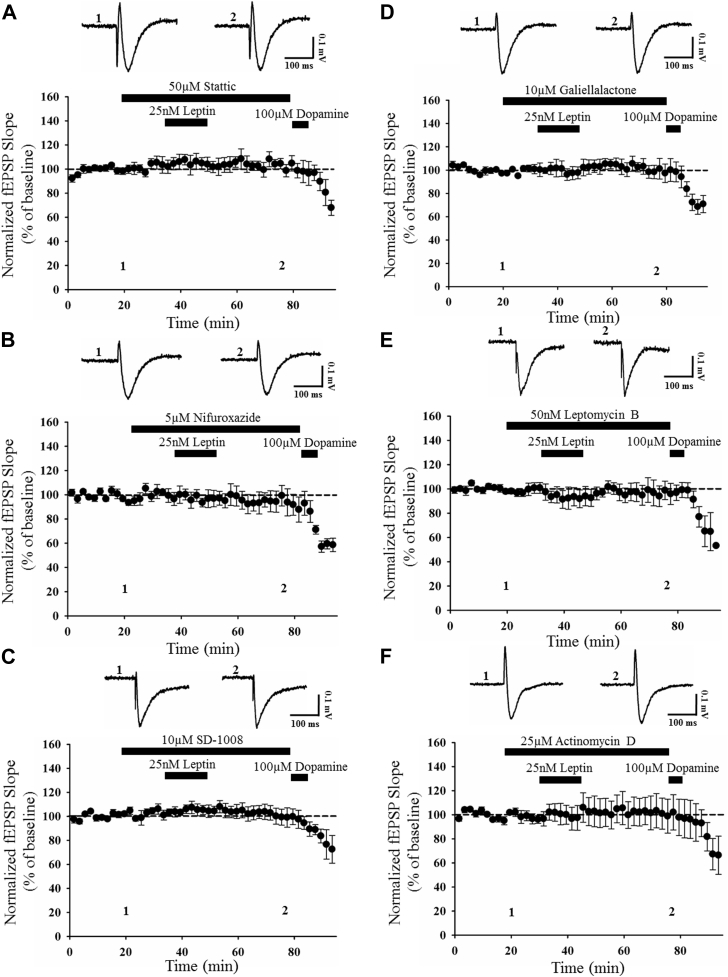
Leptin-induced LTD requires STAT3-dependent gene transcription. (A–F) Plots of pooled and normalized data illustrating the effects of leptin (25 nM) on excitatory synaptic transmission in the presence of various inhibitors. In this and subsequent figures, each point is the average of four successive responses and representative synaptic traces for each experiment are shown above each plot and for the time indicated. Treatment with the STAT3 inhibitor, stattic (50 μM; [A]); the JAK-STAT3 inhibitor, nifuroxazide (5 μM; [B]); or the JAK2-STAT3 inhibitor, SD-1008 (10 μM; [C]), all inhibited leptin-induced LTD, indicating involvement of STAT3. Likewise, inhibition of STAT3 binding to DNA (galiellalactone; 10 μM; [D]), nuclear export (leptomycin B; 50 nM; [E]), or gene transcription (actinomycin D; 25 μM; [F]) also inhibited leptin-induced LTD. Thus leptin-induced LTD involves JAK2-STAT3 signaling and subsequent gene transcription. Abbreviations: LTD, long-term depression.

A key target for JAK2 is the STAT family of transcription factors, and STAT3, in particular, is activated downstream of neuronal leptin receptors. In addition, STAT3 is highly expressed at hippocampal synapses (Murata et al., 2000), and this STAT family member is implicated in activity-dependent LTD at both SC-CA1 and TA-CA1 synapses (McGregor et al., 2017, Nicolas et al., 2012). Thus, it is feasible that STAT3 plays a role in leptin-induced LTD at TA-CA1 synapses. To address this possibility, the effects of stattic, an inhibitor of STAT3 activation, were examined (Schust et al., 2006). Application of stattic (50 μM; 40 minutes) had no effect on basal excitatory synaptic transmission (108 ± 5.3% of baseline; *n* = 4; *p* > 0.05; not shown). Moreover, in control slices, robust LTD was induced (83 ± 8.2% of baseline; *n* = 6; *p* < 0.01; not shown) by leptin (25 nM; 15 minutes). However, in interleaved slices treated with stattic, the ability of leptin to induce LTD was markedly reduced as no significant synaptic depression was observed in leptin-treated slices (104 ± 4.5% of baseline; *n* = 6; *p* > 0.05; Fig. 4A), and this effect of leptin was significantly different to leptin-induced LTD in control slices (*p* < 0.05). Together these data suggest involvement of a STAT3-dependent process in leptin-induced LTD at adult TA-CA1 synapses.

To further examine the potential role of JAK2 and STAT3 in leptin-induced LTD, the effects of nifuroxazide, a specific inhibitor of JAK-STAT3 signaling, were assessed (Nelson et al., 2008). Application of nifuroxazide (5 μM; 40 minutes) had no effect on excitatory synaptic transmission at TA-CA1 synapses (97 ± 3.2% of baseline *n* = 4; *p* > 0.05; not shown). However, the ability of leptin to induce LTD was significantly attenuated in the presence of nifuroxazide (95 ± 9.0% of baseline; *n* = 4; *p* > 0.05; Fig. 4B), compared with interleaved leptin-treated slices (*p* < 0.05) as synaptic transmission was depressed to 83 ± 8.2% of baseline (*n* = 6; *p* < 0.01; not shown) in slices treated with leptin alone. In parallel studies, the effects of SD-1008, which inhibits JAK2-STAT3 activity, were examined (Duan et al., 2007). Application of SD-1008 (10 μM; 40 minutes) had no effect on basal synaptic transmission (96 ± 2.9% of baseline *n* = 5; *p* > 0.05; not shown); however, in slices treated with SD-1008, the ability of leptin to induce LTD was inhibited as synaptic transmission was 100% ± 6.3% of baseline after application of leptin (*n* = 5; *p* > 0.05; Fig. 4C)—an effect that was significantly different to the magnitude of leptin-induced LTD in interleaved control slices (*p* < 0.05). Thus, together these data provide further evidence in support of a role for JAK2-STAT3 signaling in leptin-induced LTD at TA-CA1 synapses.

### 3.6 Leptin-induced LTD involves STAT3-driven gene transcription

Activation of the canonical JAK-STAT pathway culminates in phosphorylation and dimerization of STAT and its subsequent translocation to the nucleus where it controls gene transcription (Li, 2008). Recent studies indicate that NMDA receptor–dependent LTD evoked at adult TA-CA1 synapses requires activation of canonical JAK2-STAT3 signaling and subsequent gene transcription (McGregor et al., 2017). Thus, to examine if leptin-induced LTD involves STAT3-dependent changes in gene expression, several inhibitors that interfere with gene transcription were used. Application of galiellalactone (10 μM; 40 minutes), to inhibit STAT3 binding to DNA (Don-Doncow et al., 2014), had no effect on basal excitatory synaptic transmission (95 ± 3.0% of baseline *n* = 4; *p* > 0.05; not shown). However, in slices treated with galiellalactone, application of leptin (25 nM; 15 minutes) failed to induce LTD as synaptic transmission was 100 ± 6.7% of baseline (*n* = 4; *p* > 0.05; Fig. 4D), whereas robust LTD was induced by leptin in interleaved control slices (75 ± 10.7% of baseline; *n* = 5; *p* < 0.01; not shown). As leptin-induced LTD requires STAT3 binding to DNA, it is likely that nuclear export is also involved in this process. To address this possibility, the effects of leptomycin B, an inhibitor of nuclear signaling (Wolffe et al., 1997), were examined. Application of leptomycin B (50 nM; 40 minutes) had no effect on basal synaptic transmission (101 ± 6.5% of baseline *n* = 4; *p* > 0.05; not shown); however, it inhibited the ability of leptin to induce LTD (96 ± 8.1% of baseline; *n* = 5; *p* > 0.05; Fig. 4E). As STAT3 binding to DNA is required for gene transcription, the potential role of transcription was investigated using actinomycin D, a specific inhibitor of gene transcription (Frey et al., 1996). Application of actinomycin D (25 μM; 40 minutes) had no effect on basal synaptic transmission (97 ± 11.6% of baseline *n* = 5; *p* > 0.05; not shown), but in slices treated with actinomycin D, leptin failed to induce LTD (101 ± 41.0% of baseline; *n* = 5; *p* > 0.05; Fig. 4F). Thus, together these data indicate that STAT3 binding to DNA and subsequent gene transcriptional changes underlie the ability of leptin to induce LTD at adult TA-CA1 synapses.

### 3.7 Leptin-induced LTD involves internalization of GluA1-containing AMPA receptors

**Fig. 5:**
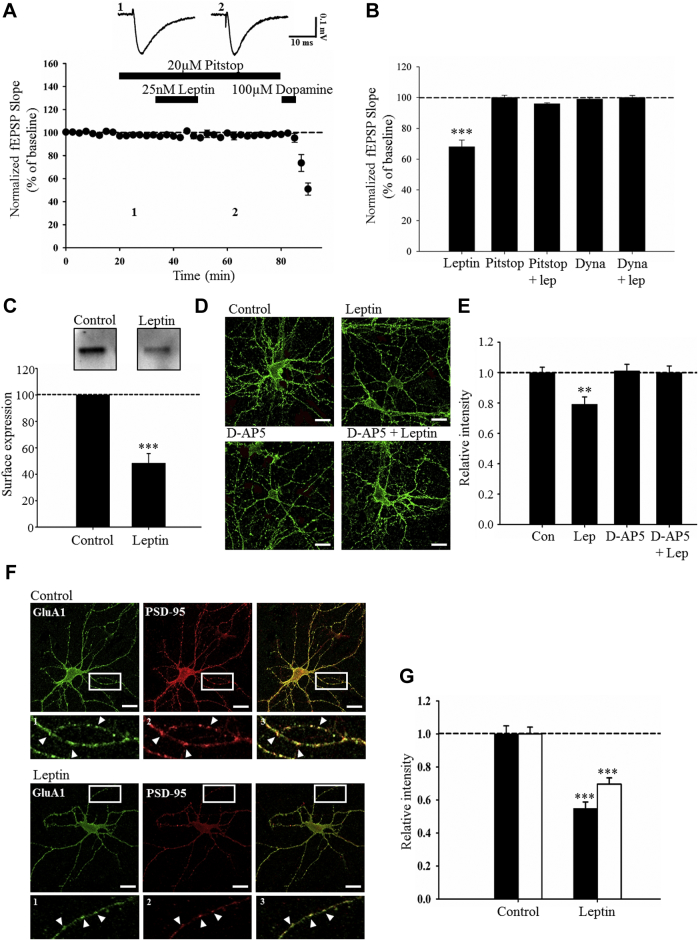
Leptin-induced LTD involves NMDA receptor–dependent internalization of GluA1. (A) Plot of pooled and normalized data illustrating the effects of leptin (25 nM) on excitatory synaptic transmission in the presence of pitstop (20 μM) to block endocytosis. Treatment with pitstop inhibited leptin-induced LTD indicating involvement of receptor endocytosis in this process. Each point is the average of four successive responses and representative synaptic traces for each experiment are shown above each plot and for the time indicated. (B) Histogram of pooled data demonstrating the relative effects of leptin (25 nM) on excitatory synaptic transmission in control conditions and in the presence of 2 distinct inhibitors of endocytosis, namely pitstop and dynamin. Blockade of endocytosis blocked the ability of leptin to induce LTD indicating a role for endocytosis. (C) Top: representative western blots of surface GluA1 expression from control and leptin (25 nM)-treated slices using cell-surface biotinylation. Bottom: corresponding histogram of GluA1 surface expression in control slices and after leptin. Leptin reduces GluA1 surface expression in adult hippocampal slices. (D) Representative confocal images of surface GluA1 immunolabeling in cultured hippocampal neurons (8–12 DIV) in control conditions (0.1 mM Mg^2+^) and after treatment with leptin (50 nM; 30 minutes), D-AP5 (50 μM; 30 minutes), and D-AP5 plus leptin. In the presence of leptin, GluA1 staining is reduced compared with control and this effect is blocked by D-AP5. In this and subsequent images, scale bars represent 20 μm. (E) Histogram of pooled data illustrating the relative intensity of GluA1 surface immunostaining in control conditions and after exposure to leptin, D-AP5, and D-AP5 plus leptin. (F) Representative confocal images of surface GluA1 (green) and PSD-95 (red) immunolabeling in control and leptin (50 nM; 30 minutes)-treated neurons. “1”, “2”, and “3” are zoomed in dendritic regions of the representative images and demonstrate co-localization of surface GluA1 and PSD-95 immunolabeling. Leptin reduces surface GluA1 and PSD-95 immunostaining relative to control. (G) Histogram of pooled data illustrating the relative intensity of GluA1 (filled bars) and PSD-95 (open bars) in control conditions and after leptin treatment. In this and subsequent figures, *, **, and *** represent *p* < 0.05, *p* < 0.01, and *p* < 0.001, respectively. Abbreviations: DIV, days in vitro; LTD, long-term depression. (For interpretation of the references to color in this figure legend, the reader is referred to the Web version of this article.)

Alterations in AMPA receptor trafficking is a key for activity-dependent synaptic plasticity (Collingridge et al., 2004), and it is known that removal of AMPA receptors from synapses underlies activity-dependent LTD (Collingridge et al., 2010). Thus, to address if AMPA receptor trafficking contributes to leptin-induced LTD, the effects of 2 specific inhibitors of endocytosis, namely dynasore (Macia et al., 2006) and pitstop (von Kleist et al., 2011) were examined. In control slices, application of leptin (25 nM; 15 minutes) resulted in persistent depression of synaptic transmission to 68 ± 4.4% of baseline (*n* = 5; *p* < 0.001; Fig. 5B). Application of either 20 μM pitstop (100 ± 1.5 % of baseline; *n* = 5; *p* > 0.05) or 100 μM dynasore (99± 1.2% of baseline *n* = 5; *p* > 0.05) had no effect on basal synaptic transmission (Fig. 5B). However, the ability of leptin to induce LTD was blocked in slices treated with either inhibitor (Fig. 5A, B), such that leptin failed to alter synaptic transmission in the presence of either pitstop (96 ± 0.6% of baseline *n* = 5; *p* > 0.05) or dynasore (100± 1.4% of baseline *n* = 5; *p* > 0.05). These data suggest that AMPA receptor endocytosis underlies leptin-induced LTD. To verify AMPA receptor endocytosis accompanies leptin-induced LTD at TA-CA1 synapses, the effects of leptin on AMPA receptor expression was also examined in hippocampal slices using cell-surface biotinylation assays (McGregor et al., 2017, Moult et al., 2010). Thus, in adult hippocampal slices treated with leptin (25 nM; 30 minutes), a significant reduction in GluA1 surface labeling (to 48 ± 7.4% of untreated control slices; *n* = 5 slices for each; *p* < 0.001) was observed (Fig. 5C), suggesting that leptin also evokes a reduction in the surface expression of GluA1-containing AMPA receptors in adult hippocampal slices.

To examine further the mechanisms underlying AMPA receptor endocytosis by leptin, the effects of leptin on the cell-surface density of AMPA receptors were also assessed using an N-terminal antibody against GluA1 on living hippocampal neurons. To model leptin-induced LTD in hippocampal neurons, all experiments were performed under conditions of enhanced excitability (0.1 mM Mg^2+^), as this increases the likelihood of leptin inducing LTD (Durakoglugil et al., 2005). In this study, application of leptin (50 nM; 30 minutes) to hippocampal neurons resulted in a significant reduction in GluA1 surface immunostaining (79 ± 4.9% of control; *n* = 36; *p* < 0.01; Fig. 5D, E). Because NMDA receptor activation promotes AMPA receptor endocytosis (Collingridge et al., 2010) and leptin-induced LTD at TA-CA1 synapses is NMDA receptor dependent, the effects of the NMDA receptor antagonist, D-AP5, were examined. Application of D-AP5 (50 μM) had no effect on surface GluA1 labeling per se (101 ± 4.3% of control; *n* = 36; *p* > 0.05; Fig. 5D, E). However, in neurons pretreated with D-AP5, the ability of leptin to reduce GluA1 surface immunolabeling was significantly reduced (100 ± 4.4% of control; *n* = 36; *p* > 0.05; Fig. 5D, E). Thus, these data indicate that the leptin-driven internalization of GluA1 involves an NMDA receptor–dependent process.

Excitatory synaptic strength typically depends on the density of AMPA receptors at synapses and removal of AMPA receptors from synapses underlies activity-dependent LTD. Thus, to determine if leptin influences the synaptic expression of GluA1-containing AMPA receptors, the distribution of surface GluA1 was compared with the synaptic marker, PSD-95, as previously (McGregor et al., 2017). Application of leptin (50 nM; 30 minutes) reduced GluA1 surface staining (55 ± 3.9% of control; *n* = 36; *p* < 0.01; Fig. 5C, D); an effect that was accompanied by a reduction in PSD-95–positive immunolabeling (70 ± 3.7% of control; *n* = 36; *p* < 0.01; Fig. 5F, G). Leptin also reduced the degree of GluA1 labeling that co-localized with PSD-95 from 58 ± 1.1% to 38 ± 3.1% co-localization (*n* = 36; *p* < 0.01). Overall, these data suggest that leptin reduces the density of GluA1-containing AMPA receptors at hippocampal synapses.

**Fig. 6:**
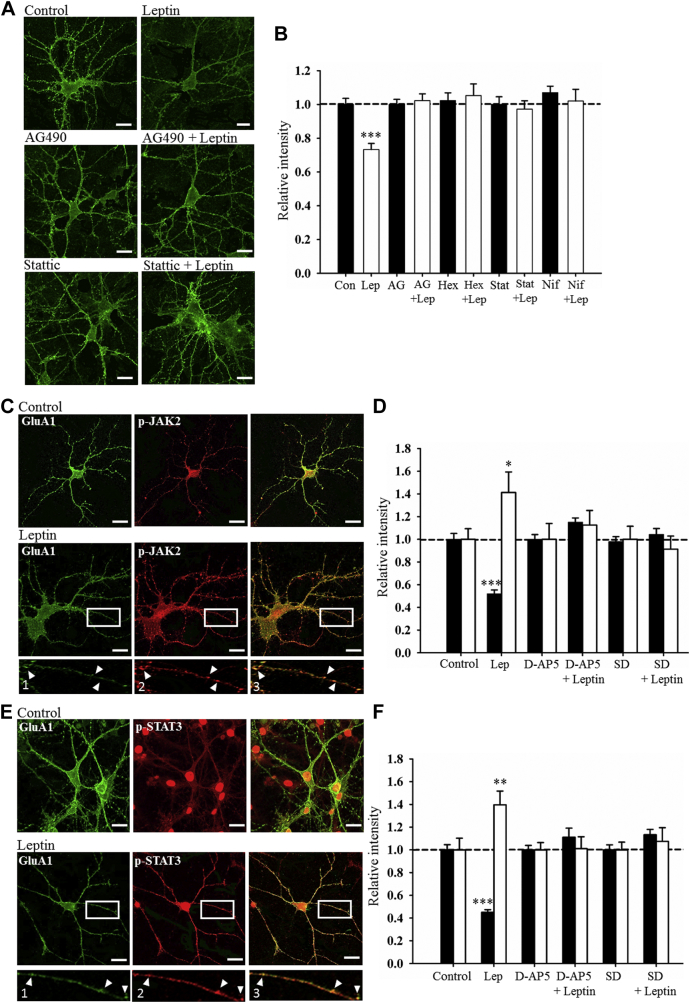
Leptin-driven internalization of GluA1 requires JAK2-STAT3 signaling. (A) Representative confocal images of surface GluA1 immunolabeling in 8–12 DIV hippocampal neurons in control conditions and after application of leptin (50 nM; 30 minutes), AG490 (10 μM), stattic (50 μM), and leptin plus AG490 or stattic. The ability of leptin to internalize GluA1 was reduced after JAK2-STAT3 inhibition. Scale bars represent 20 μM. (B) Histogram of pooled data illustrating the relative intensity of surface GluA1 in control conditions and after treatment with leptin, and in the combined presence of AG490 (10 μM), hexabromocyclohexane (50 μM), stattic (50 μM), and nifuroxazide (5 μM). Inhibition of JAK2-STAT3 signaling prevents leptin-driven GluA1 internalization in culture. (C) Representative confocal images of surface GluA1 (green) and p-JAK2 (red) in control and leptin-treated hippocampal neurons (8–12 DIV). “1”, “2”, and “3” are zoomed in dendritic regions of the representative images and show co-localization of surface GluA1 and p-JAK2 labeling. Application of leptin reduces surface GluA1 labeling and this is accompanied by increased p-JAK2 levels. (D) Histogram of pooled data showing the relative intensity of surface GluA1 (filled bars) and p-JAK2 (open bars) labeling in control conditions and after leptin (50 nM; 30 minutes), D-AP5 (50 μM), and SD-1008 (10 μM) treatment or in the presence of leptin plus D-AP5 or SD-1008. (E) Representative confocal images of surface GluA1 (green) and p-STAT3 (red) in control and leptin-treated neurons (8–12 DIV). “1”, “2”, and “3” are zoomed in dendritic regions of the representative images and show co-localization of surface GluA1 and p-STAT3 labeling. Leptin reduces GluA1 surface expression and this is accompanied by an increase in p-STAT3. (F) Histogram of pooled data indicating the relative intensity of surface GluA1 (filled bars) and p-STAT3 (open bars) labeling in control conditions, after leptin (50 nM; 30 minutes), D-AP5 (50 μM), and SD-1008 (10 μM) treatment or in the presence of leptin plus D-AP5 or SD-1008. The leptin-dependent effects on GluA1 trafficking are accompanied by an increase in JAK2 and STAT3 activity. In this and subsequent figures, *, **, and *** represent *p* < 0.05, *p* < 0.01, and *p* < 0.001, respectively. Abbreviations: DIV, days in vitro; p-JAK2, phosphorylated JAK2; p-STAT3, phosphorylated STAT3. (For interpretation of the references to color in this figure legend, the reader is referred to the Web version of this article.)

As our data indicate that JAK2-STAT3 signaling underlies leptin-induced LTD at TA-CA1 synapses, the role of this pathway in mediating the leptin-driven changes in AMPA receptor trafficking were also examined. In control neurons, treatment with leptin (50 nM; 30 minutes) reduced GluA1 surface staining (to 73 ± 3.8% of control; *n* = 36; *p* < 0.01; Fig. 6A, B). To address the general role of JAK, the effects of the broad spectrum JAK inhibitor, AG490 (10 μM) were assessed, and it had no effect on GluA1 immunostaining per se (100 ± 3.0%; *n* = 36; *p* > 0.05; Fig. 6A, B) However, treatment of hippocampal neurons with AG490 significantly inhibited the ability of leptin to reduce GluA1 surface expression (102 ± 3.9% of control; *n* = 36; *p* > 0.05; Fig. 6A, B). Selective blockade of JAK2 with hexabromocyclohexane (50 μM) also inhibited the effects of leptin as GluA1 surface labeling was 105 ± 6.9% of control in the combined presence of leptin and hexabromocyclohexane (*n* = 36; *p* > 0.05; Fig. 6B). In control neurons, treatment with hexabromocyclohexane had no effect on the surface expression of GluA1 (102 ± 4.6% of control; *n* = 36; *p* > 0.05; Fig. 6B). Together these data indicate that the JAK2 activation is required for leptin-driven alterations in AMPA receptor trafficking.

As our data indicate that leptin-induced LTD at adult TA-CA1 synapses involves STAT3 activation, the potential role of STAT3 in the leptin-driven internalization of GluA1 was also examined. In control neurons, application of the STAT3 inhibitor stattic (50 μM) or the JAK-STAT3 inhibitor nifuroxazide (5 μM) had no effect on GluA1 surface expression per se (100 ± 4.5% of control; *n* = 36; *p* > 0.05 and 100 ± 3.9% of control; *n* = 36; *p* > 0.05 respectively; Fig. 6A, B). In the absence of these inhibitors, application of leptin (50 nM; 30 minutes) reduced GluA1 surface staining to 74 ± 3.4% of control (*n* = 36; *p* < 0.001; Fig. 6A, B). However, in neurons treated with either STAT3 inhibitor, the ability of leptin to attenuate GluA1 surface immunolabeling was significantly reduced such that GluA1 surface staining was 98 ± 4.3% of control (stattic; *n* = 36; *p* > 0.05) and 102 ± 7.0% of control (nifuroxazide; *n* = 36; *p* > 0.05) (Fig. 6A, B). Together these data indicate that the ability of leptin to internalize GluA1-containing AMPA receptors requires JAK2-STAT3 signaling.

To verify further the role of JAK-STAT signaling, we determined if the internalization of GluA1 induced by leptin was associated with an increase in JAK2 activity. As JAK2 activation involves phosphorylation of tyrosine 1007–1008 (Y1007-1008) residues, dual labeling techniques were used to simultaneously compare the surface density of GluA1 and the dendritic levels of p-JAK2 (Y1007-1008) in hippocampal neurons, as before (McGregor et al., 2017). Application of leptin (50 nM; 30 minutes) reduced GluA1 immunostaining (52 ± 3.4% of control; *n* = 36; *p* < 0.001; Fig. 6C, D), which was accompanied by a significant increase in the levels of p-JAK2 (141 ± 18.2% of control; *n* = 36; *p* < 0.05; Fig. 6C, D). The leptin-driven decrease in surface GluA1 expression and concomitant increase in p-JAK2 immunolabeling required NMDA receptor activation as blockade of NMDA receptors with D-AP5 (50 μM) inhibited both processes (GluA1; 115 ± 3.8% of control; *n* = 36; *p* > 0.05 and p-JAK2; 112 ± 5.3% of control; *n* = 36; *p* > 0.05; Fig. 6D). Furthermore, application of SD-1008, to inhibit JAK2-STAT3 signaling also attenuated the leptin-driven reduction in GluA1 surface expression (104 ± 11.5% of control; *n* = 36; *p* > 0.05; Fig. 6D) and increase in p-JAK2 (91 ± 11.5% of control; *n* = 36; *p* > 0.05; Fig. 6D). Together these data not only further verify a role for JAK2-STAT3 signaling in leptin-induced TA-CA1 LTD, but also indicate that NMDA receptor activation is pivotal for the leptin-driven reduction in GluA1 surface expression and increase in JAK2 activity.

It is known that JAK2 phosphorylation promotes dimerization and phosphorylation of STAT3 monomers. As STAT3 dimerization requires phosphorylation of tyrosine 705 (Y705), we also assessed if leptin alters STAT3 activity by simultaneously comparing GluA1 surface immunostaining and p-STAT3 levels in hippocampal neurons (McGregor et al., 2017). Application of leptin evoked a significant reduction in GluA1 surface staining (45 ± 2.3% of control; *n* = 36; *p* < 0.001; Fig. 6E, F); an effect that was accompanied by an increase in p-STAT3 (to 140 ± 12.1% of control; *n* = 36; *p* > 0.05; Fig. 6E, F). The leptin-driven decrease in GluA1 surface labeling (111 ± 8.1% of control; *n* = 36; *p* > 0.05; Fig. 6F) and simultaneous increase in p-STAT3 activity (101 ± 10.4%; *n* = 36; *p* > 0.05; Fig. 6F) were inhibited by D-AP5 (50 μM), indicating involvement of NMDA receptors. Moreover, inhibition of canonical JAK2-STAT3 signaling with SD-1008 (10 μM) prevented the effects of leptin on GluA1 trafficking (113 ± 4.8% of control; *n* = 36; *p* > 0.05; Fig. 6F) and p-STAT3 (107 ± 12.0% of control; *n* = 36; *p* > 0.05; Fig. 6F). Overall, these data indicate that the leptin-driven internalization of GluA1-containing AMPA receptors is accompanied by an increase in JAK2 and STAT3 activity and that these effects of leptin are NMDA receptor dependent.

### 3.8 Activity-dependent LTD and leptin-induced LTD share similar expression mechanisms

**Fig. 7:**
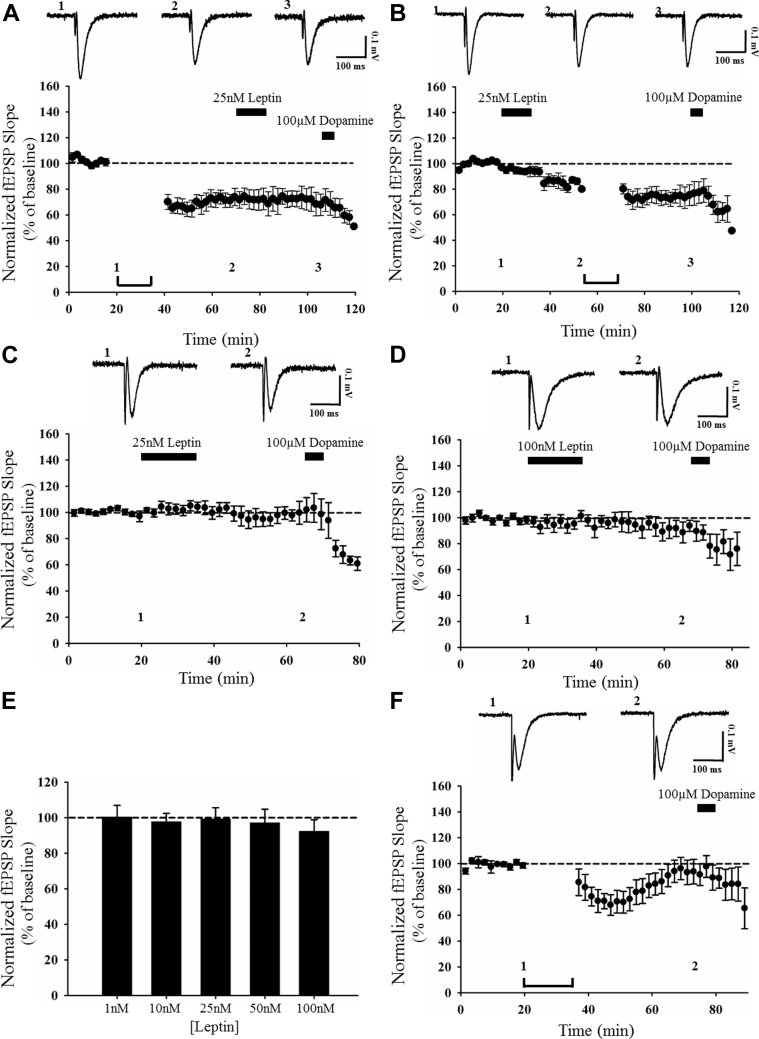
Leptin-induced LTD and activity-dependent LTD share similar expression mechanisms; however, this changes with aging. (A, B) Plots of pooled and normalized data indicating that LFS (1 Hz, 900 shocks; 15 minutes) induces LTD; however, subsequent addition of leptin (25 nM; 15 minutes) fails to alter the magnitude of LFS-induced LTD (A). In this and subsequent figures, each point is the average of four successive responses and representative synaptic traces for each experiment are shown above each plot and for the time indicated. Application of LFS after induction of leptin-induced LTD also fails to significantly alter synaptic transmission (B). (C) Plot of pooled and normalized data highlighting that LFS fails to induce LTD in aged (12–14 months old) hippocampus at TA-CA1 synapses. (D, E) Plot of pooled and normalized data showing that 25 nM (D) and 100 nM (E) leptin fail to alter synaptic transmission in aged hippocampus. (F) Histogram illustrating that application of a range of leptin concentrations (1 nM, 10 nM, 25 nM, 50 nM, 100 nM; 15 minutes) has no effect on synaptic transmission at aged TA-CA1 synapses. Abbreviations: LTD, long-term depression; LFS, low-frequency stimulation.

Leptin-induced LTD at adult TA-CA1 synapses displays similarities to activity-dependent LTD induced at the same synapse, as both processes are NMDA receptor dependent and involve JAK2-STAT3 signaling (McGregor et al., 2017). In view of this, it is feasible that both forms of LTD share similar expression mechanisms. To address this, occlusion experiments were performed. In the first series of experiments, an LFS (1 Hz; 900 shocks, 15 minutes) paradigm was initially delivered to induce LTD (McGregor et al., 2017), which resulted in a significant synaptic depression (to 71 ± 2.8% of baseline; *n* = 5; *p* < 0.001; Fig. 7A). Subsequent addition of leptin (25 nM; 15 minutes) after 20 minutes of stable LFS-induced LTD, did not significantly alter synaptic transmission (*p* > 0.05), as synaptic transmission remained depressed (73 ± 3.5% baseline; *n* = 5; *p* < 0.001; Fig. 7A). Conversely, prior application of leptin (25 nM; 15 minutes) resulted in a persistent depression of synaptic transmission (84 ± 2.9% of baseline; *n* = 4; *p* < 0.001; Fig. 7B), and the magnitude of leptin-induced LTD was not significantly altered (74 ± 5.5% of baseline; *n* = 4; *p* < 0.001; Fig. 7B) by subsequent application of LFS 20 minutes after leptin washout (*p* > 0.05). Consequently, as leptin-induced LTD is occluded by LFS-induced LTD and vice versa at adult TA-CA1 synapses, this suggests that leptin-induced LTD shares a common mechanism of expression with LFS-induced LTD at this synapse.

### 3.9 Age-dependent alterations in leptin regulation of synaptic transmission at TA-CA1 synapses

Previous studies have shown that neuronal responsiveness to leptin declines with age, which may be due to age-related alterations in leptin receptor expression and/or signaling (Scarpace et al., 2000). Indeed, our previous studies indicate that the ability of leptin to induce LTP at SC-CA1 synapses is significantly attenuated in aged (12–14 months old), compared with adult (12–16 weeks old), hippocampus (Moult and Harvey, 2011). Thus, to assess if there are similar age-dependent changes in leptin responsiveness at TA-CA1 synapses, the effects of leptin were examined in aged (12–14 months old) hippocampal slices. Unlike adult hippocampus, application of leptin (25 nM; 15 minutes) failed to alter basal synaptic transmission (99 ± 6.3% of baseline; *n* = 8; *p* > 0.05; Fig. 7C, E) in aged slices. As leptin can alter excitatory synaptic transmission over a range of concentrations (Luo et al., 2015, Moult et al., 2010, Shanley et al., 2001), the effects of different concentrations of leptin were evaluated. However, application of either 1 nM leptin (100 ± 6.5% of baseline; *n* = 5; *p* > 0.05; Fig. 7E) and 10 nM leptin (98 ± 4.7% of baseline; *n* = 5; *p* > 0.05; Fig. 7E) had no significant effect on basal synaptic transmission at TA-CA1 synapses. Higher concentrations of leptin were also examined as we have shown that higher concentrations of leptin are required to induce LTP at juvenile TA-CA1 synapses (Luo et al., 2015). However, addition of either 50 nM leptin (97 ± 7.6% of baseline; *n* = 5; *p* > 0.05; Fig. 7E) or 100 nM leptin (92 ± 6.5% of baseline; *n* = 6; *p* > 0.05) also failed to evoke any significant change in excitatory synaptic transmission at aged TA-CA1 synapses (Fig. 7D, E). These data suggest that in contrast to its actions in adult hippocampus, leptin has no effect on excitatory synaptic transmission at TA-CA1 synapses in aged hippocampus.

As similar expression mechanisms underlie leptin-induced LTD and activity-dependent LTD at TA-CA1 synapses, we explored if age-related changes also occur in the ability of LFS to induce LTD in aged hippocampus. Previous studies and this study indicate that LTD is readily induced at adult TA-CA1 synapses using a standard LFS paradigm (Dvorak-Carbone and Schuman, 1999, McGregor et al., 2017). However, in contrast to adult hippocampus, application of an LFS (1 Hz; 900 shocks, 15 minutes) paradigm also failed to evoke LTD in aged hippocampus such that synaptic transmission was 92 ± 8.6% of baseline after LFS (*n* = 7; *p* > 0.05; Fig. 7F).

**Fig. 8:**
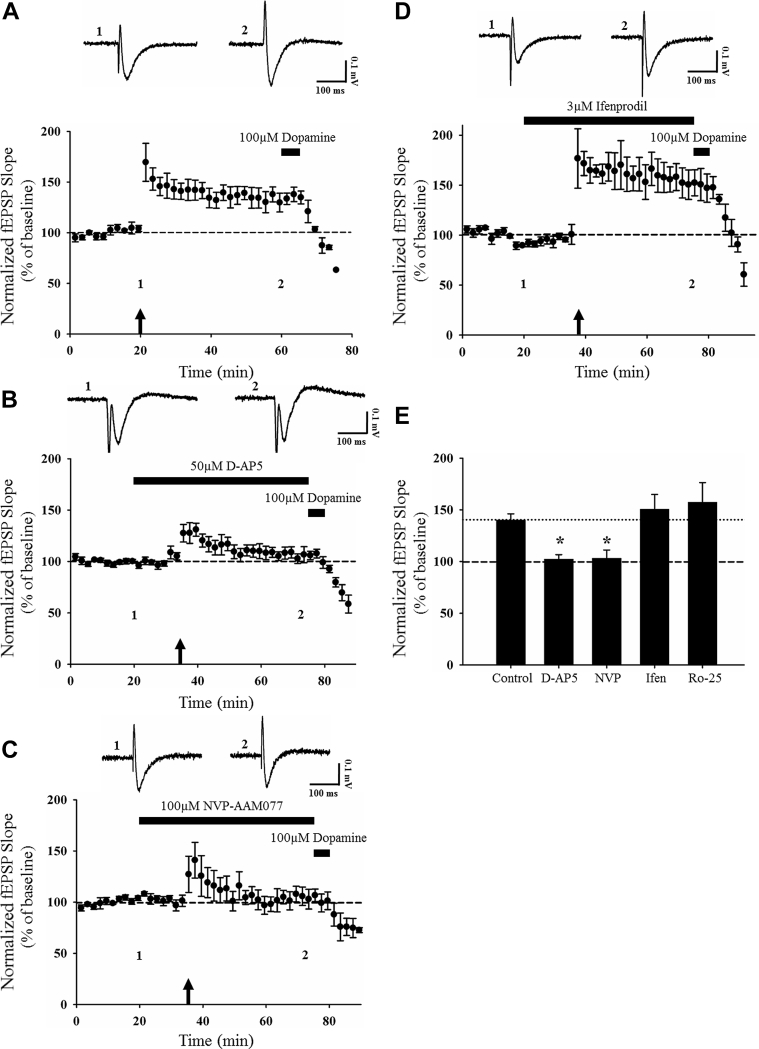
Activity-dependent LTP at aged TA-CA1 synapses requires activation of GluN2A-containing NMDA receptors. (A-D) Plots of pooled and normalized data demonstrating that high-frequency stimulation (HFS; 100 Hz, 1 second) induces LTP at TA-CA1 synapses in aged hippocampal slices (A); however, LTP is blocked in the presence of D-AP5 (50 μM; [B]) and NVP-AAM077 (100 μM; [C]) but not ifenprodil (3 μM; [D]). Thus, HFS-induced LTP requires selective activation of GluN2A-containing NMDA receptors. In this and subsequent figures, each point is the average of four successive responses and representative synaptic traces for each experiment are shown above each plot and for the time indicated. (E) Histogram of pooled data illustrating the magnitude of HFS-induced LTP in control conditions and in presence of the NMDA receptor antagonist D-AP5 (50 μM), the GluN2A inhibitor NVP-AAM077 (100 μM), and the GluN2B inhibitors, ifenprodil (3 μM) and Ro-256981 (3 μM). In this figure, *, **, and *** represent *p* < 0.05, *p* < 0.01, and *p* < 0.001, respectively. Abbreviations: LTP, long-lasting increase.

As leptin-induced LTD and activity-dependent LTD at TA-CA1 synapses are both NMDA receptor–dependent processes, it is feasible that age-related alterations in NMDA receptor function occurs that renders aged TA-CA1 synapses unresponsive to the activity-driven changes that are required for LTD induction. To assess this possibility, we examined if other forms of activity-dependent synaptic plasticity are intact at aged TA-CA1 synapses. In this respect, we examined if application of a HFS paradigm (100 Hz, 1 second) resulted in the induction of LTP, as our previous studies indicate that HFS readily induces NMDA receptor–dependent LTP at juvenile and adult TA-CA1 synapses (Luo et al., 2015, McGregor et al., 2017). Application of HFS induced robust LTP at TA-CA1 synapses such that synaptic transmission was increased to 140 ± 6.0% of baseline (*n* = 5; *p* < 0.001; Fig. 8A) in aged hippocampal slices. To verify that HFS-induced LTP displays NMDA receptor dependence as before (Luo et al., 2015), the effects of the NMDA receptor antagonist, D-AP5 (50 μM) were investigated. Application of D-AP5 had no effect on basal synaptic transmission (100 ± % 10.5 of baseline; *n* = 5; *p* > 0.05; not shown); however, D-AP5 did block the ability of HFS to induce LTP (102 ± 4.3% of baseline; *n* = 6; *p* > 0.05; Fig. 8B, E). This effect was significantly different (*p* < 0.05; Fig. 8E) to the magnitude of LTP evoked in interleaved control slices (133 ± 8.8% of baseline; *n* = 5; *p* < 0.001; Fig. 8E).

As molecularly distinct NMDA receptor subunits underlie activity-dependent hippocampal synaptic plasticity (Bartlett et al., 2007, Liu et al., 2004) and leptin-driven changes in synaptic transmission at both SC-CA1 and TA-CA1 synapses require distinct NMDA receptor GluN2 subunits (Luo et al., 2015, Moult and Harvey, 2011), the role of different GluN2 subunits in HFS-induced LTP was addressed. Application of the putative GluN2A antagonist, NVP-AMM077 had no effect on basal excitatory synaptic transmission (92 ± 8.2% of baseline; *n* = 4; *p* > 0.05; not shown) but it blocked the ability of HFS to induce LTP (103 ± 8.0% of baseline; *n* = 6; *p* > 0.05; Fig. 8C, E), suggesting that activity-dependent LTP in aged hippocampus requires activation of GluN2A receptor subunits. To verify a selective role for GluN2A, the effects of GluN2B antagonists were also examined. Application of 2 distinct GluN2B inhibitors had no effect on basal synaptic transmission (ifenprodil; 92 ± 9.2% of baseline; *n* = 5; *p* > 0.05 and Ro-256981; 96 ± 7.5% of baseline; *n* = 5; *p* > 0.05). However, inhibition of GluN2B had no effect on activity-dependent LTP as HFS readily induced LTP in the presence of either ifenprodil (151 ± 14.4% of baseline; *n* = 5; *p* < 0.01; Fig. 8D, E), or Ro-256981 (157 ± 19.0% of baseline; *n* = 5; *p* < 0.001; Fig. 8E). There was no significant difference between the magnitude of LTP evoked in control conditions (140 ± 6.0% of baseline; *n* = 5; *p* < 0.001; Fig. 8E) and LTP induced in the presence of either ifenprodil or Ro-256981 (*p* > 0.05 for each; Fig. 8E). Overall, these data indicate that HFS-induced LTP at aged hippocampal synapses requires selective activation of GluN2A-containing NMDA receptors.

In conclusion, these data suggest that there are marked alterations in the sensitivity of TA-CA1 synapses to leptin with age, as the ability of leptin to regulate excitatory synaptic transmission is completely absent in aged hippocampus. In addition, the ability of leptin and LFS to induce LTD is also absent at aged TA-CA1 synapses, indicating that the ageing process also markedly influences the ability of this synapse to undergo LTD.

## 4 Discussion

Evidence is growing that the central actions of the endocrine hormone leptin extend beyond its actions in the hypothalamus and the regulation of food intake and body weight (Harvey, 2013). Indeed, numerous studies indicate that leptin is a potent modulator of hippocampal excitatory synaptic function (Irving and Harvey, 2014). In this respect, leptin is reported to have potential cognitive enhancing properties as it facilitates the cellular events underlying hippocampal-dependent learning and memory, including effects on synaptic plasticity and glutamate receptor trafficking at hippocampal SC-CA1 synapses (Moult et al., 2010, Moult and Harvey, 2011). However, leptin also markedly influences excitatory synaptic transmission at the anatomically distinct TA input to CA1 pyramidal neurons as leptin induces a novel form of LTP at juvenile TA-CA1 synapses (Luo et al., 2015). In this study, we provide the first compelling evidence that leptin also regulates excitatory synaptic transmission at adult, but not aged, TA-CA1 synapses. In contrast to its actions in juvenile hippocampus, leptin induces a novel form of LTD in adulthood: a phenomenon that shares similar expression mechanisms to activity-dependent LTD evoked at adult TA-CA1 synapses (McGregor et al., 2017). Moreover, in a manner similar to NMDA receptor–dependent LTD at SC-CA1 synapses (Nicolas et al., 2012), JAK-STAT signaling is pivotal for leptin-induced LTD at adult TA-CA1 synapses. However, unlike NMDA receptor–dependent LTD at SC-CA1 synapses (Nicolas et al., 2012), STAT3-dependent changes in gene transcription underlie leptin-induced LTD at adult TA-CA1 synapses.

The ability of leptin to induce LTD at TA-CA1 synapses is likely to involve a postsynaptic expression mechanism as leptin-driven synaptic depression was not linked to any change in the PPR. However, the possibility that a presynaptic event contributes to leptin-induced LTD cannot be excluded as change in PPR does not entirely exclude a presynaptic form of LTD. Leptin-induced LTD required activation of NMDA receptors, as the ability of leptin to induce LTD was completely blocked in slices exposed to the competitive NMDA receptor antagonist, D-AP5. Leptin-induced LTD was independent of GABA_A_ or GABA_B_ receptor activation as pharmacological blockade of GABAergic inhibition had no effect on the magnitude of LTD. Moreover, mechanistically leptin-induced LTD at TA-CA1 synapses displays parallels to other forms of hippocampal LTD as NMDA receptor activation and a postsynaptic mechanism of expression also underlies activity-dependent LTD at adult TA-CA1 synapses (McGregor et al., 2017) and juvenile SC-CA1 synapses (Collingridge et al., 2010). Furthermore, in this study, we found that leptin-induced LTD at TA-CA1 synapses and activity-dependent LTD evoked at the same synapse share similar expression mechanisms as LFS-induced LTD occluded leptin-induced LTD and vice versa.

Previous studies indicate that NMDA receptor activation is crucial for the effects of leptin on excitatory synaptic transmission at SC-CA1 synapses (Moult and Harvey, 2011, Moult et al., 2010, Oomura et al., 2006). Indeed, the ability of leptin to induce LTP, reverse LTP, and induce LTD at SC-CA1 synapses all involve the synaptic activation of NMDA receptors (Irving and Harvey, 2014). NMDA receptor activation is also required for leptin-driven changes in glutamate receptor trafficking (Moult et al., 2010). Recent studies have demonstrated that molecularly distinct NMDA receptors are necessary for different forms of activity-dependent hippocampal synaptic plasticity (Bartlett et al., 2007, Liu et al., 2004). In accordance with this, leptin-induced LTD was observed in slices treated with 2 distinct inhibitors of GluN2B suggesting the involvement of a GluN2B-independent mechanism. Moreover, blockade of GluN2A subunits with the putative GluN2A antagonist, NVP-AAM077, prevented the effects of leptin, indicating that leptin-induced LTD is likely to involve activation of GluN2A, but not GluN2B, subunits. Although previous studies have shown that it is difficult to discern GluN2A-dependent effects in acute brain slices (Frizelle et al., 2006), and it is known that NVP-AAM077 displays limited selectivity for GluN2A over GluN2B subunits (Bartlett et al., 2007), the possibility that NVP-AAM007 has a small effect on GluN2B subunits cannot be excluded in this study. However, as 2 distinct inhibitors of GluN2B subunits failed to reduce the magnitude of leptin-induced LTD at TA-CA1 synapses, activation of GluN2B subunits is unlikely to be required for leptin to induce LTD. This contrasts with activity-dependent LTD at hippocampal SC-CA1 synapses that is reported to involve activation of GluN2B-containing NMDA receptors (Bartlett et al., 2007, Liu et al., 2004) and more recently GluN2B di-heretromers (France et al., 2017).

In contrast to the present study, the novel form of LTP induced by leptin at juvenile TA-CA1 synapses involves selective activation of GluN2B-containing NMDA receptors (Luo et al., 2015). This suggests that distinct NMDA receptors are required for the divergent effects on synaptic efficacy induced by leptin at TA-CA1 synapses at different ages. These findings also indicate that the polarity of leptin's effects on synaptic efficacy at the TA input to CA1 neurons is highly dependent on age. In a similar manner, the direction of leptin's effect on excitatory synaptic efficacy at SC-CA1 synapses is also age dependent, and distinct NMDA receptor subunits are implicated in the bidirectional age-related effects of leptin at hippocampal SC-CA1 synapses (Moult and Harvey, 2011).

Previous studies have identified a role for JAK signaling in activity-dependent LTD at juvenile SC-CA1 synapses (Nicolas et al., 2012), and adult TA-CA1 synapses (McGregor et al., 2017). Here, we provide evidence to support JAK involvement in leptin-induced LTD at adult TA-CA1 synapses, as the ability of leptin to induce LTD was blocked by the general inhibitor of JAK signaling, AG490. Selective activation of JAK2 is implicated in leptin-induced LTD as pharmacological inhibition of JAK2 prevented the induction of LTD by leptin. The proposed role of JAK2 parallels its involvement in other forms of synaptic plasticity as activation of JAK2 has been shown to underlie NMDA receptor–dependent LTD at SC-CA1 synapses (Nicolas et al., 2012) and adult TA-CA1 synapses (McGregor et al., 2017). It is known that STAT3 is the prominent STAT activated by neuronal leptin receptors, and thus, it is likely that STAT3 is the STAT isoform that is activated downstream of JAK2. Our data support this as selective inhibitors of STAT3 blocked the ability of leptin to induce LTD in adult hippocampal slices. Moreover, broad spectrum inhibitors of JAK2-STAT3 signaling also prevented leptin-induced LTD at TA-CA1 synapses. Thus, together these data indicate that activation of the JAK2-STAT3 pathway is required for leptin-induced LTD at adult hippocampal TA-CA1 synapses.

Trafficking of AMPA receptors to and away from synapses is pivotal for hippocampal activity-dependent synaptic plasticity (Collingridge et al., 2004), and endocytosis of synaptic AMPA receptors is key for activity-dependent LTD at hippocampal SC-CA1 synapses (Collingridge et al., 2010). As leptin regulates glutamate receptor trafficking processes (Moult et al., 2010), it is feasible that leptin-driven alterations in AMPA receptor trafficking is required for leptin-induced LTD at TA-CA1 synapses. Indeed, our data indicate that removal of AMPA receptors from synapses is crucial for leptin-induced LTD as under conditions that enable leptin-induced LTD (Durakoglugil et al., 2005), as treatment with selective inhibitors of endocytosis blocked the ability of leptin to induce LTD at adult hippocampal TA-CA1 synapses. Furthermore, exposure of cultured hippocampal neurons to leptin resulted in a reduction of GluA1 surface expression and the density of GluA1-containing AMPA receptors at synapses. Moreover, the ability of leptin to promote AMPA receptor removal was blocked after inhibition of JAK2 or STAT3 signaling. Furthermore, leptin-induced alterations in AMPA receptor trafficking were accompanied by increases in the levels of both p-JAK2 and p-STAT3 indicating that the activity of JAK2 and STAT3 is increased after leptin-induced LTD and AMPA receptor internalization.

Previous studies have identified a role for noncanonical JAK-STAT signaling in NMDA receptor–dependent LTD as activity-dependent LTD induced at SC-CA1 synapses is independent of STAT3-dependent gene transcription (Nicolas et al., 2012). By contrast, here we show that gene transcriptional changes are pivotal for leptin-induced LTD at TA-CA1 synapses as inhibition of STAT3-dependent transcription prevented the induction of LTD by leptin. The involvement of gene transcriptional changes in this study parallels the role of gene transcription that underlies activity-dependent LTD at adult TA-CA1 synapses (McGregor et al., 2017). Previous studies also implicate gene transcriptional changes in LTD, as rapid elevations in immediate early genes have been detected after LTD induction in adult hippocampus (Lindecke et al., 2006). In addition, inhibitors of mRNA and protein synthesis have also been shown to block the induction of LTD at juvenile SC-CA1 synapses (Kauderer and Kandel, 2000).

In this study, application of leptin had no effect on excitatory synaptic transmission at aged TA-CA1 synapses, which contrasts with leptin's effects in adulthood. Previous studies have shown that aged hippocampal slices display reduced responsiveness to leptin compared with adult tissue (Moult and Harvey, 2011). Moreover, decreases in STAT3 activity combined with increased expression of regulators that limit leptin receptor signaling have been reported in aged tissue (Scarpace et al., 2000). All of these factors may contribute to the lack of leptin effect at aged TA-CA1 synapses. However, LFS also failed to induce LTD at aged TA-CA1 synapses, suggesting that the ability of TA-CA1 synapses to undergo LTD is lost during the ageing process. The inability of either leptin or LFS to induce LTD at aged TA-CA1 synapses contrasts with aged SC-CA1 synapses where robust LTD is readily induced by LFS (Foster and Kumar, 2007). The age-related alterations in the capacity of TA-CA1 synapses to undergo persistent changes in synaptic efficacy appears to be restricted to the process of LTD as robust activity-dependent LTP was observed at aged TA-CA1 synapses. This form of TA-CA1 LTP requires activation of GluN2A-containing, but not GluN2B-containing NMDA receptor subunits. As this parallels the involvement of GluN2A subunits in leptin-induced LTD at adult TA-CA1 synapses, it is unlikely that significant age-related alterations in the density and/or localization of GluN2A subunits occurs and thus contribute to the failure of leptin or activity to induce LTD at aged TA-CA1 synapses.

### 4.1 Physiological significance

Although the functional significance of LTD induced by leptin at adult TA-CA1 synapses is not yet known, it is feasible that leptin-induced LTD plays a role in novelty detection that is linked to episodic memory formation. In this respect, the direct TA input to CA1 neurons is implicated in spatial novelty detection as well as episodic memory processes (Remondes and Schuman, 2004, Suh et al., 2011, Vago et al., 2007). In support of this possibility, recent studies indicate that exposure to leptin enhances performance in episodic-like memory tasks in rodents (Malekizadeh et al., 2017), whereas hyperleptinemia has been linked to increased novelty-seeking behaviors in adult rats (Fraga-Marques et al., 2009). It is well known that circulating leptin levels are linked to body fat content (Zhang et al., 1994), and leptin levels are elevated in obese individuals resulting in development of leptin resistance. Obesity-related leptin resistance is associated with impairments in hippocampal-dependent learning and memory in rodents (Li et al., 2002) and in humans (Paz-Filho et al., 2008). Recent studies have also identified deficits in episodic memory in obese adults (Cheke et al., 2016), suggesting that dysfunctions in the leptin system result in impaired episodic memory function. Thus, the ability of leptin to induce LTD at hippocampal TA-CA1 synapses may be important for episodic memory not only in health but also in diseases linked to resistance or insensitivity to leptin.

Neurodegenerative disorders such as AD result in impairments in cognitive function and loss of memory. Age is a key risk factor for AD; however, lifestyle and diet are also important contributory factors. Clinical evidence has revealed a significantly greater risk of developing AD in individuals with midlife obesity, suggesting that impaired leptin function or leptin resistance contributes to the incidence of AD. In support of this, reduced circulating levels of leptin is a common feature in AD patients (Power et al., 2001) and in rodent models of AD (Farr et al., 2006), which has fueled the possibility that the leptin system may be therapeutically useful in age-related disorders such as AD. Indeed, recent studies indicate that leptin prevents the detrimental effects of Aβ on hippocampal synaptic function in cellular models of AD (Doherty et al., 2013, Malekizadeh et al., 2017).

It is known that the EC is a highly vulnerable brain region, and it is one of the first areas to degenerate in AD. Indeed, phosphorylated tau accumulates in all layers of the EC, before widespread accumulation in the hippocampus. Histological studies have shown that the TA input, which originates in the EC, is particularly vulnerable, and it exhibits early structural and synaptic deficits in AD models (Yassa et al., 2010). Moreover, in tauopathy models of AD, significant alterations in synaptic plasticity have been detected at TA-CA1 synapses (Booth et al., 2016). Thus, as leptin dysfunction has been linked to ageing and AD, and leptin regulates a synaptic connection that is highly susceptible to degeneration, the ability of leptin to regulate synaptic efficacy at TA-CA1 synapses is likely to have important implications for age-related neurodegenerative disorders such as AD.

## Disclosure statement

The authors have no potential or actual conflicts of interest.

## References

[bib1] Aksoy-Aksel A., Manahan-Vaughan D. (2013). The temporoammonic input to the hippocampal CA1 region displays distinctly different synaptic plasticity compared to the Schaffer collateral input in vivo: significance for synaptic information processing. Front Synaptic Neurosci..

[bib2] Anderson W.W., Collingridge G.L. (2007). Capabilities of the WinLTP data acquisition program extending beyond basic LTP experimental functions. J. Neurosci. Methods..

[bib3] Bartlett T.E., Bannister N.J., Collett V.J., Dargan S.L., Massey P.V., Bortolotto Z.A., Fitzjohn S.M., Bashir Z.I., Collingridge G.L., Lodge D. (2007). Differential roles of NR2A and NR2B-containing NMDA receptors in LTP and LTD in the CA1 region of two-week old rat hippocampus. Neuropharmacology.

[bib4] Booth C.A., Witton J., Nowacki J., Tsaneva-Atanasova K., Jones M.W., Randall A.D., Brown J.T. (2016). Altered intrinsic pyramidal neuron properties and pathway-specific synaptic dysfunction underlie aberrant hippocampal network function in a mouse model of tauopathy. J. Neurosci..

[bib5] Braak E., Braak H. (1997). Alzheimer’s disease: transiently developing dendritic changes in pyramidal cells of sector CA1 of the Ammon’s horn. Acta. Neuropathol..

[bib6] Cheke L.G., Simons J.S., Clayton N.S. (2016). Higher body mass index is associated with episodic memory deficits in young adults. Q. J. Exp. Psychol. (Hove).

[bib7] Collingridge G.L., Peineau S., Howland J.G., Wang Y.T. (2010). Long-term depression in the CNS. Nat. Rev. Neurosci..

[bib8] Collingridge G.L., Isaac J.T.R., Wang Y.T. (2004). Receptor trafficking and synaptic plasticity. Nat. Rev. Neurosci..

[bib9] Doherty G.H., Beccano-Kelly D., Yan S.D., Gunn-Moore F.J., Harvey J. (2013). Leptin prevents hippocampal synaptic disruption and neuronal cell death induced by amyloid β. Neurobiol. Aging.

[bib10] Don-Doncow N., Escobar Z., Johansson M., Kjellström S., Garcia V., Munoz E., Sterner O., Bjartell A., Hellsten R. (2014). Galiellalactone is a direct inhibitor of the transcription factor STAT3 in prostate cancer cells. J. Biol. Chem..

[bib11] Duan Z., Bradner J., Greenberg E., Mazitschek R., Foster R., Mahoney J., Seiden M.V. (2007). 8-benzyl-4-oxo-8-azabicyclo[3.2.1]oct-2-ene-6,7-dicarboxylic acid (SD-1008), a novel janus kinase 2 inhibitor, increases chemotherapy sensitivity in human ovarian cancer cells. Mol. Pharmacol..

[bib12] Durakoglugil M., Irving A.J., Harvey J. (2005). Leptin induces a novel form of NMDA receptor-dependent long-term depression. J. Neurochem..

[bib13] Dvorak-Carbone H., Schuman E.M. (1999). Long-term depression of temporoammonic-CA1 hippocampal synaptic transmission. J. Neurophysiol..

[bib14] Farr S.A., Banks W.A., Morley J.E. (2006). Effects of leptin on memory processing. Peptides.

[bib15] Fischer G., Mutel V., Trube G., Malherbe P., Kew J.N., Mohacsi E., Heitz M.P., Kemp J.A. (1997). Ro 25-6981, a highly potent and selective blocker of N-Methyl-D-aspartate receptors containing the NR2B subunit. Characterization in vitro. J. Pharmacol. Exp. Ther..

[bib16] Foster T.C., Kumar A. (2007). Susceptibility to induction of long-term depression is associated with impaired memory in aged Fischer 344 rats. Neurobiol. Learn. Mem..

[bib17] Frey U., Frey S., Schollmeier F., Krug M. (1996). Influence of actinomycin D, a RNA synthesis inhibitor, on long-term potentiation in rat hippocampal neurons in vivo and in vitro. J. Physiol..

[bib18] Frizelle P.A., Chen P.E., Wyllie D.J. (2006). Equilibrium constants for (R)-[(S)-1-(4-bromo-phenyl)-ethylamino]-(2,3-dioxo-1,2,3,4-tetrahydroquinoxalin-5-yl)-methyl]-phosphonic acid (NVP-AAM077) acting at recombinant NR1/NR2A and NR1/NR2B N-methyl-D-aspartate receptors: implications for studies of synaptic transmission. Mol. Pharmacol..

[bib19] Fraga-Marques M.C., Moura E.G., Claudio-Neto S., Trevenzoli I.H., Toste F.P., Passos M.C., Lisboa P.C., Manhães A.C. (2009). Neonatal hyperleptinaemia programmes anxiety-like and novelty seeking behaviours but not memory/learning in adult rats. Horm. Behav..

[bib20] France G., Fernández-Fernández D., Burnell E.S., Irvine M.W., Monaghan D.T., Jane D.E., Bortolotto Z.A., Collingridge G.L., Volianskis A. (2017). Multiple roles of GluN2B-containing NMDA receptors in synaptic plasticity in juvenile hippocampus. Neuropharmacology.

[bib57] Harvey J. (2013). Leptin regulation of neuronal morphology and hippocampal synaptic function. Front Synaptic Neurosci.

[bib21] Irving A.J., Harvey J. (2014). Leptin regulation of hippocampal synaptic function in health and disease. Philos Trans R Soc Lond. B Biol Sci.

[bib22] Kauderer B.S., Kandel E.R. (2000). Capture of a protein synthesis-dependent component of long-term depression. Proc. Natl. Acad. Sci. U S A.

[bib58] Li X.L., Aou S., Oomura Y., Hori N., Fukunaga K., Hori T. (2002). Impairment of long-term potentiation and spatial memory in leptin receptor-deficient rodents. Neurosci.

[bib23] Lindecke A., Korte M., Zagrebelsky M., Horejschi V., Elvers M., Widera D., Prullage M., Pfeiffer J., Kaltschmidt B., Kalschmidt C. (2006). Long-term depression activates transcription of immediate early transcription factor genes: involvement of serum response factor/Elk-1. Eur. J. Neurosci..

[bib24] Liu L., Wong T.P., Pozza M.F., Lingenhoehl K., Wang Y., Sheng M., Auberson Y.P., Wang Y.T. (2004). Role of NMDA receptor subtypes in governing the direction of hippocampal synaptic plasticity. Science.

[bib25] Li W.X. (2008). Canonical and non-canonical JAK-STAT signalling. Trends. Cell. Biol..

[bib26] Luo X., McGregor G., Irving A.J., Harvey J. (2015). Leptin induces a novel form of NMDA receptor-dependent LTP at hippocampal temporoammonic-CA1 synapses. eNeuro.

[bib27] Macia E., Ehrlich M., Massol R., Boucrot E., Brunner C., Kirchhausen T. (2006). Dynasore, a cell-permeable inhibitor of dynamin. Dev. Cell..

[bib28] Malekizadeh Y., Holiday A., Redfearn D., Ainge J.A., Doherty G., Harvey J. (2017). A leptin fragment mirrors the cognitive enhancing and neuroprotective actions of leptin. Cereb. Cortex..

[bib29] McGregor G., Harvey J. (2017). Leptin regulation of synaptic function at hippocampal TA-CA1 and SC-CA1 synapses: implications for health and disease. Neurochem. Res..

[bib30] McGregor G., Irving A.J., Harvey J. (2017). Canonical JAK-STAT signalling is pivotal for long-term depression at adult hippocampal temporoammonic-CA1 synapses. FASEB J..

[bib31] Meydan N., Grunberger T., Dadi H., Shahar M., Arpaia E., Lapidot Z., Leeder J.S., Freedman M., Cohen A., Gazit A., Levitzki A., Roifman C.M. (1996). Inhibition of acute lymphoblastic leukaemia by a Jak-2 inhibitor. Nature.

[bib32] Moult P.R., Cross A., Santos S.D., Carvalho A.L., Lindsay Y., Connolly C.N., Irving A.J., Leslie N.R., Harvey J. (2010). Leptin regulates AMPA receptor trafficking via PTEN inhibition. J. Neurosci..

[bib33] Moult P.R., Harvey J. (2011). NMDA receptor subunit composition determines the polarity of leptin-induced synaptic plasticity. Neuropharmacology.

[bib34] Murata S., Usuda N., Okano A., Kobayashi S., Suzuki T. (2000). Occurrence of a transcription factor, signal transducer and activators of transcription 3 (Stat3), in the postsynaptic density of the rat brain. Brain. Res. Mol. Brain. Res..

[bib35] Nakashiba T., Young J.Z., McHugh T.J., Buhl D.L., Tonegawa S. (2008). Transgenic inhibition of synaptic transmission reveals role of CA3 output in hippocampal learning. Science.

[bib36] Nelson E.A., Walker S.R., Kepich A., Gashin L.B., Hideshima T., Ikeda H., Chauhan D., Anderson K.C., Frank D.A. (2008). Nifuroxazide inhibits survival of multiple myeloma cells by directly inhibiting STAT3. Blood.

[bib37] Nicolas C.S., Peineau S., Amici M., Csaba Z., Fafouri A., Javalet C., Collett V.J., Hildebrandt L., Seaton G., Choi S.L., Sim S.E., Bradley C., Lee K., Zhuo M., Kaang B.K., Gressens P., Dournaud P., Fitzjohn S.M., Bortolotto Z.A., Cho K., Collingridge G.L. (2012). The JAK/STAT pathway is involved in synaptic plasticity. Neuron.

[bib38] Oomura Y., Hori N., Shiraishi T., Fukunaga K., Takeda H., Tsuji M., Matsumiya T., Ishibashi M., Aou S., Li X.L., Kohno D., Uramura K., Sougawa H., Yada T., Wayner M.J., Sasaki K. (2006). Leptin facilitates learning and memory performance and enhances hippocampal CA1 long-term potentiation and CAMK II phosphorylation in rats. Peptides.

[bib39] Otmakhova N.A., Lisman J.E. (1999). Dopamine selectively inhibits the direct cortical pathway to the CA1 hippocampal region. J. Neurosci..

[bib40] Paz-Filho G.J., Babikian T., Asarnow R., Delibasi T., Esposito K., Erol H.K., Wong M.L., Licinio J. (2008). Leptin replacement improves cognitive development. PLoS One.

[bib41] Power D.A., Collins R., Neill O. (2001). Circulating leptin levels and weight loss in Alzheimer’s disease patients. Dement. Geriatr. Cogn. Disord..

[bib42] Remondes M., Schuman E.M. (2004). Role for a cortical input to hippocampal area CA1 in the consolidation of a long-term memory. Nature.

[bib43] Sandberg E.M., Ma X., He K., Frank S.J., Ostrov D.A., Sayeski P.P. (2005). Identification of 1,2,3,4,5,6-hexabromocyclohexane as a small molecular inhibitor of JAK2 tyrosine kinase autophoshorylation. J. Med. Chem..

[bib44] Scarpace P.J., Matheny M., Moore R.L., Tümer N. (2000). Impaired leptin responsiveness in aged rats. Diabetes.

[bib45] Schust J., Sperl B., Hollis A., Mayer T.U., Berg T. (2006). Stattic: a small-molecule inhibitor of STAT3 activation and dimerization. Chem. Biol..

[bib46] Shanley L.J., Irving A.J., Harvey J. (2001). Leptin enhances NMDA receptor function and modulates hippocampal synaptic plasticity. J. Neurosci..

[bib47] Shanley L.J., O’Malley D., Irving A.J., Ashford M.L.J., Harvey J. (2002). Leptin inhibits epileptiform-like activity in rat hippocampal neurones via PI 3-kinase-driven activation of BK channels. J. Physiol..

[bib48] Spiegelman B.M., Flier J.S. (2001). Obesity and the regulation of energy balance. Cell.

[bib49] Suh J., Rivest A.J., Nakashiba T., Tominaga T., Tonegawa S. (2011). Entorhinal cortex layer III input to the hippocampus is crucial for temporal association memory. Science.

[bib50] Vago D.R., Bevan A., Kesner R.P. (2007). The role of the direct perforant path input to the CA1 subregion of the dorsal hippocampus in memory retention and retrieval. Hippocampus.

[bib51] von Kleist L., Stahlschmidt W., Bulut H., Gromova K., Puchkov D., Robertson M.J., MacGregor K.A., Tomilin N., Pechstein A., Chau N., Chircop M., Sakoff J., von Kries J.P., Saenger W., Kräusslich H.G., Shupliakov O., Robinson P.J., McCluskey A., Haucke V. (2011). Role of the clathrin terminal domain in regulating coated pit dynamics revealed by small molecule inhibition. Cell.

[bib52] Wayner M.J., Armstrong D.L., Phelix C.F., Oomura Y. (2004). Orexin-A (Hypocretin-1) and leptin enhance LTP in the dentate gyrus of rats in vivo. Peptides.

[bib53] Williams K. (1993). Ifenprodil discriminates subtypes of the N-methyl-D-aspartate receptor: selectivity and mechanisms at recombinant heteromeric receptors. Mol. Pharmacol..

[bib54] Wolff B., Sanglier J.J., Wang Y. (1997). Leptomycin B is an inhibitor of nuclear export: inhibition of nucleo-cytoplasmis translocation of the human immunodeficiency virus type 1 (HIV-1) rev protein and Rev-dependent mRNA. Chem. Biol..

[bib55] Yassa M., Muftuler L.T., Stark C.E.L. (2010). Ultrahigh-resolution microstructural diffusion tensor imaging reveals perforant path degradation in aged humans in vivo. Proc. Natl. Acad. Sci. U S A.

[bib56] Zhang Y., Proenca R., Maffei M., Barone M., Leopold L., Friedman J.M. (1994). Positional cloning of the mouse obese gene and its human homologue. Nature.

